# Diabetes and Hyperglycemia Affect Platelet GPIIIa Expression: Effects on Adhesion Potential of Blood Platelets from Diabetic Patients under In Vitro Flow Conditions

**DOI:** 10.3390/ijms21093222

**Published:** 2020-05-02

**Authors:** Tomasz Przygodzki, Boguslawa Luzak, Hassan Kassassir, Ewelina Mnich, Magdalena Boncler, Karolina Siewiera, Marcin Kosmalski, Jacek Szymanski, Cezary Watala

**Affiliations:** 1 Department of Haemostasis and Haemostatic Disorders, Chair of Biomedical Sciences, Medical University of Lodz, Mazowiecka 6/8, 92-215 Lodz, Poland; boguslawa.luzak@umed.lodz.pl (B.L.); hassan.kassassir1@gmail.com (H.K.); mnich.ewelina@gmail.com (E.M.); magdalena.boncler@umed.lodz.pl (M.B.); ksiewiera@gmail.com (K.S.); cezary.watala@umed.lodz.pl (C.W.); 2 Department of Cytobiology and Proteomics, Chair of Biomedical Sciences, Medical University of Lodz, Mazowiecka 6/8, 92-215 Lodz, Poland; 3 Department of Clinical Pharmacology, Medical University of Lodz, Kopcinskiego 22, 90-153 Lodz, Poland; marcin.kosmalski@umed.lodz.pl; 4 Central Scientific Laboratory, Medical University of Lodz, Mazowiecka 6/8, 92-215 Lodz, Poland; jacek.szymanski@umed.lodz.pl

**Keywords:** diabetes, platelets, adhesion, fibrinogen, von Willebrand factor, glycoprotein IIIa

## Abstract

Blood platelets play a crucial role in the early stages of atherosclerosis development. The process is believed to require firm adhesion of platelets to atherosclerosis-prone sites of the artery. However, little evidence exists regarding whether the blood platelets of individuals with pathological conditions associated with atherosclerosis have higher potential for adhesion. This process is to a large extent dependent on receptors present on the platelet membrane. Therefore, the aim of the presented study was to determine whether blood platelets from diabetic patients have higher capacity of adhesion under flow conditions and how diabetes affects one of the crucial platelet receptors involved in the process of adhesion—GPIIIa. The study compares the ability of platelets from non-diabetic and diabetic humans to interact with fibrinogen and von Willebrand factor, two proteins found in abundance on an inflamed endothelium, under flow conditions. The activation and reactivity of the blood platelets were also characterized by flow cytometry. Platelets from diabetic patients did not demonstrate enhanced adhesion to either studied protein, although they presented increased basal activation and responsiveness towards low concentrations of agonists. Platelets from diabetic patients were characterized by lower expression of GPIIIa, most likely due to an enhanced formation of platelet-derived microparticles PMPs, as supported by the observation of elevated concentration of this integrin and of GPIIIa-positive PMPs in plasma. We conclude that altered functionality of blood platelets in diabetes does not increase their adhesive potential. Increased glycation and decrease in the amount of GPIIIa on platelets may be partially responsible for this effect. Therefore, higher frequency of interactions of platelets with the endothelium, which is observed in animal models of diabetes, is caused by other factors. A primary cause may be a dysfunctional vascular wall.

## 1. Introduction

The development of atherosclerosis is a complex process, in which several factors are involved, including the dysfunction of endothelial layer, inflammatory cytokines and enhanced recruitment of leukocytes. Understanding the contribution of each of these components is important for identifying novel anti-atherosclerotic therapies. In recent years, it has been found that blood platelets play an important role in the formation of atherosclerotic plaques. Activated platelets have been shown to induce pro-adhesive phenotype of endothelium [1]. This facilitated the recruitment of monocytes to the vascular wall and their migration to the subendothelial space [2,3]. It was suggested that these events are preceded by a firm adhesion of platelets to atherosclerosis-prone sites of the artery [4]. Since pathological conditions associated with atherosclerosis, such as diabetes, are also characterized by increased blood platelet reactivity, it could be assumed that increased adhesion potential is another hallmark of blood platelets in these conditions. However, there is a lack of evidence indicating that blood platelets in individuals with such pathological conditions have higher potential for adhesion. We have recently shown that blood platelets in diabetic mice adhere more frequently to the intact endothelium in vivo [5]. However, blood platelets from these mice ex vivo were not interacting with vWF (von Willebrand factor) under flow conditions more frequently that those from non-diabetic mice. This suggested that the increased frequency of interactions observed in vivo was not due to abnormal platelets. On the other hand, the model of diabetes used in these studies did not fully mimic human diabetes. Therefore, we aimed to verify this hypothesis with the use of human platelets from diabetic patients. The main goal of the presented study was therefore to verify whether blood platelets from diabetic patients demonstrate increased potential to firm adhesion when compared to non-diabetic patients. This study is focused on the events which precede the formation of atherosclerotic plaque, i.e., when blood flow conditions are not yet disturbed by narrowed lumen of the vessel and the subendothelial matrix is not exposed. Therefore, substrates for platelet adhesion in this study consist of either fibrinogen or vWF, two main proteins found on an inflamed endothelium, and flow conditions represent these in an average normal artery. The study also evaluates how diabetes affects one of the crucial platelet receptors involved in the process of adhesion—GPIIIa. Selected hallmarks of blood platelet activation and reactivity were assessed with the use of flow cytometry in order to characterize the functional status of circulating blood platelets. It also examines whether exposure of non-diabetic platelets to hyperglycemic conditions would affect their adhesion potential.

## 2. Results

### 2.1. Adhesion, Activation and Reactivity of Blood Platelets from Diabetic and Non-Diabetic Individuals

**Figure 1:**
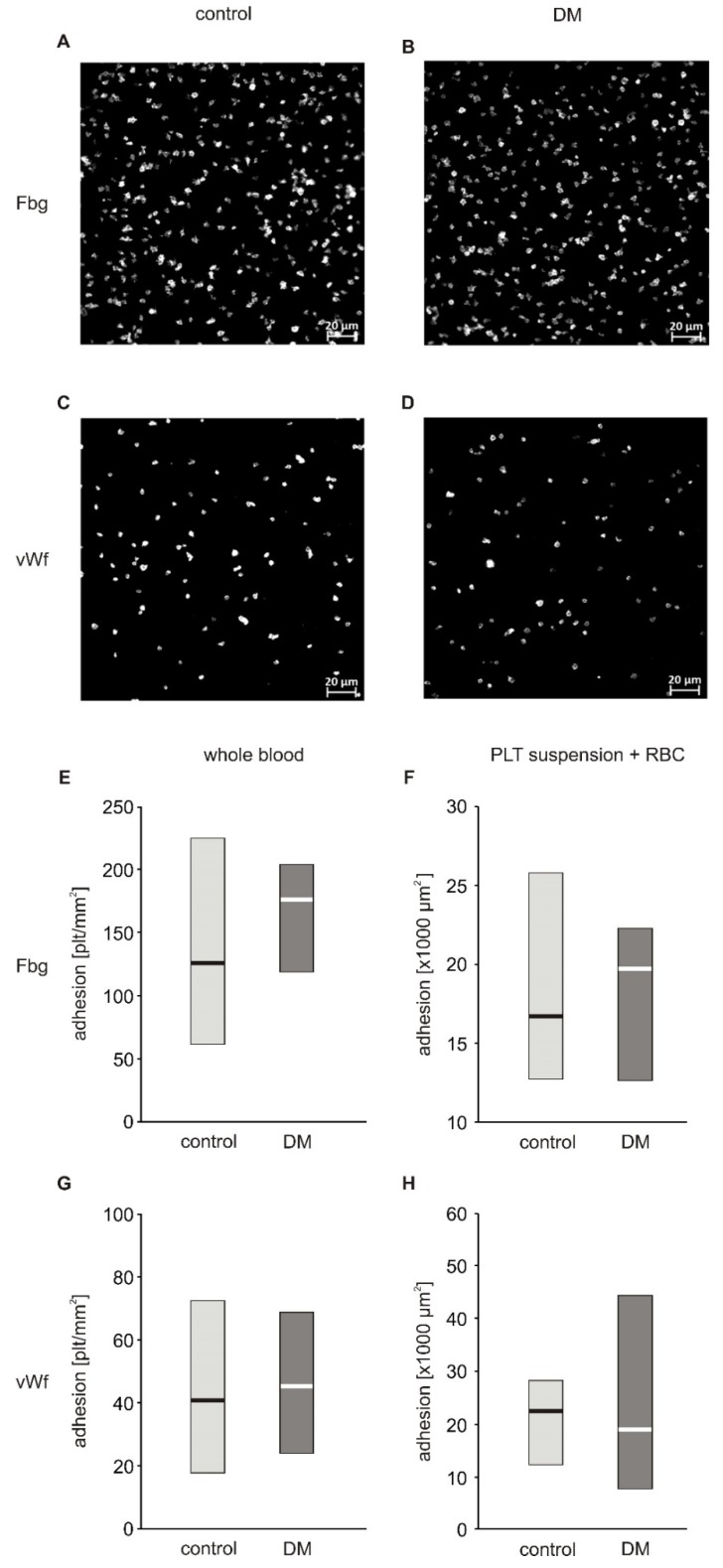
Adhesion of blood platelets from type 2 diabetic patients under flow conditions. Results presented as median (horizontal line) and interquartile range (box). Exemplary pictures showing adhesion of blood platelets from non-diabetic (**A**,**C**) and diabetic patients (**B**,**D**) to fibrinogen (**A**,**B**) and vWF (**C**,**D**). Adhesion of blood platelets to fibrinogen (**E**,**F**) and von Willebrand factor (vWF) (**G**,**H**) assayed in whole blood diluted with autologous platelet poor plasma (*n* = 17–21) (**E**,**G**) and in platelet suspension in Tyrode buffer containing autologous erythrocytes (*n* = 8) (**F**,**H**). Mean age 49.7 ± 7.8 (control) vs. 56.3 ± 8.8 (DM) (mean ± SD). Results are expressed as a number of platelets per surface area (**E**,**G**) and as area covered by platelets (**F,H**). More experimental details are given in the *Materials and Methods* section. Statistical significance of differences between the group of diabetic patients and control subjects, estimated with the non-paired Student’s t-test, was: adhesion in whole blood: n.s., control > DM; adhesion in platelet suspension: n.s., control > DM.

In a first approach to evaluate the adhesive properties of platelets to fibrinogen and vWF, we used whole blood diluted with autologous platelet poor plasma to reach a final platelet count of 100,000 plt/µL. Platelet count normalization was performed to decrease inter-patient variability. The degree of firm adhesion of blood platelets to fibrinogen (Fg) and to vWF in whole blood diluted with autologous PPP did not significantly differ between diabetic and non-diabetic patients (Figure 1E,G). The area of individual platelets spread on fibrinogen (19.20 (17.43; 19.89) µm^2^) in the non-diabetic group and (18.80 (17.54; 19.60) µm^2^) in diabetic patients as well as that spread on vWF (16.32 (15.68; 17.56) µm^2^) in non-diabetic and (16.80 (16.35; 18.76) µm^2^) in diabetic patients did not differ significantly (Figure S2 and S3). Correlation analysis revealed that adhesion to both Fg and vWF was closely associated with hematocrit: adhesion to Fg (R*s* = 0.721, *p* = 0.019) and adhesion to vWF (Rs = 0.723, *p* = 0.018). Diluting whole blood with plasma in this protocol resulted in a decrease in hematocrit. Based on this observation, and on published data showing that adhesion is strongly affected by hematocrit when its values fall below 40% [6,7], we modified the protocol to assure normalization of both platelet count and hematocrit. Isolated blood platelets suspended in Tyrode’s buffer with normalized counts were combined with autologous RBC to achieve a hematocrit of 50%. The actual hematocrit values obtained were in the range of 40%–50% and hence the effect of hematocrit is negligible. This suspension was run at the same shear stress as whole blood in the previous protocol. In these conditions, no differences between diabetic and non-diabetic blood were observed (Figure 1F,H). In the first protocol, platelets adhered separately to each other and the results of the first protocol are therefore presented as the number of platelets per surface area (Figure 1E,G). In the second protocol, in addition to platelets which adhered as single objects, a fraction of platelets formed clusters in which individual platelets were undistinguishable (Figure S4). Therefore, the results of the second protocol are expressed as area covered by platelets (Figure 1F,H). Since a fraction of platelets formed clusters, the platelets located on top of the cluster were not quantified, which may be considered as underestimation of the total area covered by platelets. However, since the aim of the experiment was to quantify platelets’ adhesion to substrate proteins and not their aggregation, excluding of these platelets from calculus did not affect the conclusions.

**Figure 2:**
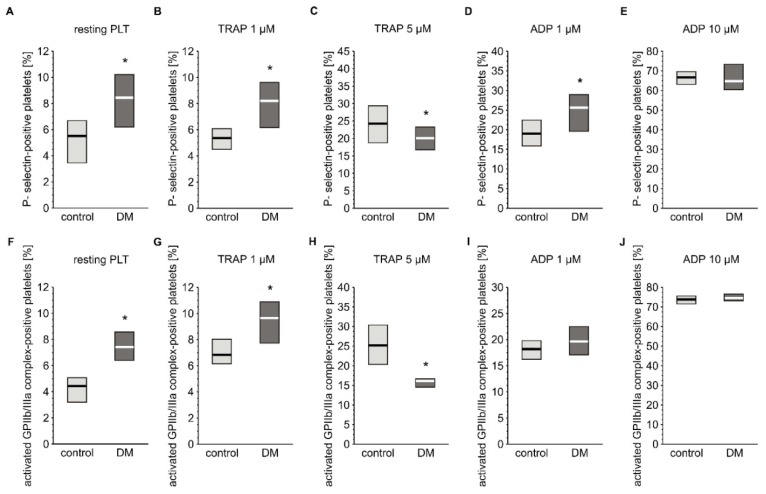
Expression of CD62P and the active form of GPIIb/IIIa on platelets from type 2 diabetic patients. Results are presented as median (vertical line) and interquartile range (box) (*n* = 35 for each group). Mean age 55.0 ± 8.1 (control) vs. 58.2 ± 7.4 (DM) (mean ± SD). The activation of circulating platelets (**A**,**F**) and platelets reactivity in response to 1 µM (**B**,**G**) or 5 µM TRAP (**C**,**H**) and 1 µM (**D,I**) or 10 µM ADP (**E**,**J**) was estimated using flow cytometry in diabetic patients and control subjects on the basis of the surface platelet expression of P-selectin (**A**–**E**) and the activated GPIIb/IIIa (PAC-1 binding) (**F**–**J**). More experimental details are given in the *Materials and Methods* section. The asterisk denotes the particular significances estimated with the use of one-tailed Mann–Whitney U test using bootstrap-boosted analyses: CD62P expression: * *p* < 0.001, control_resting_ < DM_resting_; *p* < 0.001, control_TRAP 1 µM_ < DM_TRAP 1 µM_; *p* < 0.01, control_TRAP 5 µM_ > DM_TRAP 5µM_; *p* < 0.01, control_ADP 1 µM_ < DM _ADP 1 µM_; activated GPIIb/IIIa expression: *p* < 0.001, control_resting_ < DM_resting_; *p* < 0.001, control_TRAP 1 µM_ < DM_TRAP 1 µM_; *p* < 0.001, control_TRAP 5 µM_ > DM_TRAP 5µM._

Basal activation status and reactivity of blood platelets was evaluated by flow cytometry based on the expression of their markers. The reactivity of platelets was assessed by measuring their response to agonists such ADP or TRAP. Concentration of soluble markers of platelet activation in plasma were also assayed. Expression of P-selectin and the active form of GPIIb/IIIa was slightly but significantly elevated in resting platelets from diabetic individuals (Figure 2A,F). Surprisingly, concentration of plasma-soluble P-selectin was lower in diabetic patients (3.03 (0.00; 6.50) ng/mL) compared to non-diabetic individuals (6.28 (0.65; 14.1) ng/mL) (median; IQR, *p* < 0.05, *n* = 36 (non-DM) and *n* = 39 (T2DM)). Blood platelets from T2DM patients treated with 1 µM TRAP had higher expression of P-selectin than those of non-diabetic patients, but absolute values where comparable with resting platelets; this implies that this concentration of TRAP did not evoke any response in platelets in either group (Figure 2B). In turn, 5 µM TRAP increased the expression of P-selectin in both groups; however, P-selectin expression was significantly lower in the diabetic group than in the non-diabetic group (Figure 2C). Stimulation of blood platelets with 1 µM ADP significantly increased expression of P-selectin in the diabetic group (Figure 2D). In turn, 10 µM ADP caused equally high expression of P-selectin in both groups (Figure 2E). Greater expression of the active form of GPIIb/IIIa was observed in diabetic than in non-diabetic individuals following treatment with 1 µM TRAP (Figure 2G), while 5 µM TRAP had the opposite effect (Figure 2H). ADP increased the expression of the active form of GPIIb/IIIa to the same extent in T2DM and non-diabetic groups at both tested concentrations (Figure 2I,J).

Analysis of the exogenous fibrinogen binding over time (2.5, 5, 7.5, 10, and 15 min after the addition of fibrinogen) showed that shortly (2.5 min) after adding fibrinogen to the blood sample, non-stimulated diabetic platelets had a lower capacity to bind fibrinogen (the fraction of fibrinogen bound-platelets was 2.5% ± 1.5% for T2DM, and 4.7% ± 3.0% for control, *p* = 0.064, *n* = 7). This difference vanished with prolonged times of observation, thus the fraction of platelets positive for bound fibrinogen reached up to 30% at 15 min for both groups. The addition of agonists such as ADP (0.1 µM) or TRAP (1 µM) increased fibrinogen binding to platelets similarly for both groups (the fraction of fibrinogen bound-platelets enhanced 3-fold or 2-fold after ADP or TRAP adding, respectively).

**Figure 3:**
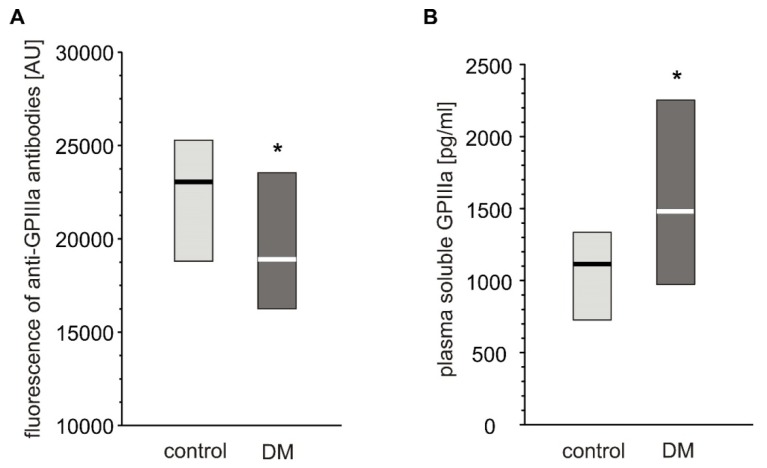
Level of GPIIIa on platelet surface and in blood plasma from type 2 diabetic patients and control subjects. Results presented as median (vertical line) and interquartile range box. Expression of GPIIIa on platelets surface was assayed by flow cytometry (*n* = 33) (**A**); concentration of soluble GPIIIa in plasma was determined by ELISA (n_control_ = 36, n_DM_ = 41). Mean age 53.9 ± 8.0 (control) vs. 57.3 ± 7.7 (DM) (mean ± SD) (**B**). More experimental details are given in the *Materials and Methods* section. The asterisk denotes the particular significances estimated with non-paired Student’s t-test: GPIIIa expression: * *p* < 0.05, control > DM; plasma-soluble GPIIIa concentration: *p* < 0.01, control < DM.

Expression of GPIIIa on platelets from T2DM patients was significantly lower than in non-diabetic individuals (Figure 3A). However, the level of plasma-soluble GPIIIa in plasma was significantly elevated in T2DM patients compared to non-diabetic individuals (Figure 3B).

To assess whether a decrease in platelet GPIIIa levels was associated with an increase in GPIIIa-positive objects (platelet-derived microparticles, PMP), we assayed their count in plasma of diabetic and non-diabetic individuals. T2DM subjects demonstrated a higher number of PMPs smaller than 0.5 µm in plasma compared to controls (Table 1).

Interestingly, the number of particles in the range of 0.5–1 µm constituted less than 10% of all MPs. To better reflect the heterogeneity in platelet size and in the abundance of GPIIIa on the platelet surface, overall platelet population was decomposed into smaller subpopulations, as briefly described in the section ‘*Statistical Analysis*’. Upon the differentiation of overall platelet population into the size-dependent subpopulations, it appeared that diabetic patients demonstrated a larger population of smaller platelets compared to healthy individuals (*p* < 0.05, estimated with bootstrap-boosted Student’s t-test), while the opposite was observed for the population of larger platelets (*p* < 0.05, estimated with the bootstrap-boosted Student’s t-test). Moreover, the fraction of platelet aggregates was found to be increased in patients with diabetes, compared to the control group (*p* < 0.05, estimated with the bootstrap-boosted Mann–Whitney U test). Platelets from diabetic patients were characterized by decreased expression of GPIIIa for both platelet subpopulation 1 and subpopulation 2 (i.e., subpopulations with small and high number of GPIIIa copies, respectively), compared to the control (*p* < 0.05, estimated with the bootstrap-boosted Student’s t-test). Interestingly, for diabetic patients, subpopulation 1 was greater than control values, while subpopulation 2 was smaller (*p* < 0.05 for both comparisons, estimated with the bootstrap-boosted Student’s t-test). In diabetic platelets, the variation in the number of GPIIIa copies in subpopulation 2 was smaller than in control platelets (*p* < 0.05, estimated with the bootstrap-boosted Student’s t-test).

### 2.2. Glycation of Platelet Membrane Proteins

To assess the effects of hyperglycemia on the platelet protein components, membrane protein glycation was assessed by the borohydrate method. Membrane protein glycation was significantly higher in platelets of T2DM patients: (4.72 ± 1.11 nmol glucose/mg protein) compared to non-diabetic individuals (3.64 ± 0.88 nmol glucose/mg, *p* < 0.01, *n* = 15).

**Figure 4:**
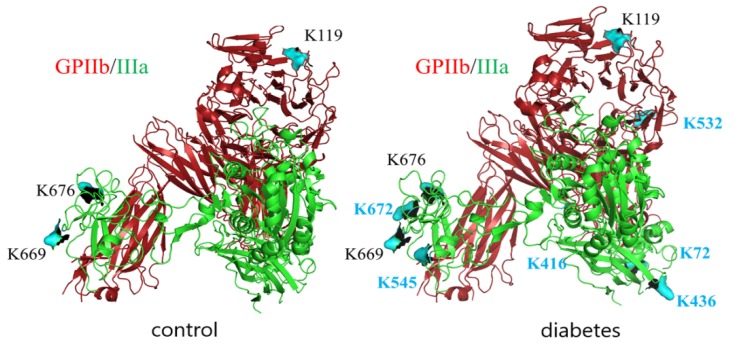
Glycation sites in GPIIb/IIIa isolated from platelet membranes of non-diabetic and diabetic individuals revealed by LC-MS/MS analysis. GPIIb is presented in red, GPIIIa is presented in green, lysin residues which were found to be modified by glucose in control samples are depicted in black, and those which were found to be modified only in diabetic samples are depicted in blue.

Since GPIIb/IIIa is a most abundant protein in platelet membranes and it plays a crucial role in adhesion to both fibrinogen and vWF, the extent of glycation-related modifications in this protein was explored in more detail. LC-MS/MS analysis of GPIIb/IIIa isolated from T2DM patients and control subjects was performed to identify the glycation sites of this integrin In both groups, integrin was glycated; however, the number of glycated lysines identified was higher in T2DM patients (residues K119 in subunit GPIIb and K669, K676, K764, K774 in subunit GPIIIa in control vs. K119, K532, K1025 in subunit GPIIb and K72, K436, K545, K645, K669, K676, K672, K764, K774 in subunit GPIIIa for T2DM) (Figure 4).

To confirm whether the number of identified glycation sites falls in the range of the sites accessible for glucose, the number of glucose molecules which can bind to GPIIb/IIIa molecule in near-physiological conditions was evaluated. In vitro glycation of purified (commercial) GPIIb/IIIa with ^14^C-glucose has shown that 18.6 ± 2.25 nmol of glucose was bound to 1 nmol of the protein.

### 2.3. In Vitro Effects of Hyperglycemia on Blood Platelets’ Adhesion Potential and GPIIIa Expression

**Figure 5:**
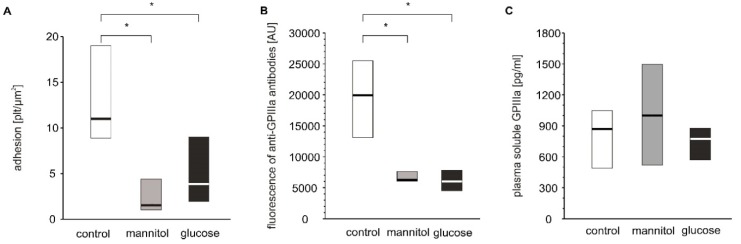
In vitro effects of high glucose concentrations on platelet adhesion, platelet surface membrane abundance of GPIIIa and plasma-soluble GPIIIa. Results presented as median (horizontal line) and interquartile range (box); *n* = 9. Mean age of 28.9 ± 10.6 years (mean ± SD). Adhesion of platelets to fibrinogen (**A**), CD61 (GPIIIa) expression on platelet surface presented as fluorescence median (**B**) and concentration of sCD61 (soluble GPIIIa) in blood plasma (**C**) were assayed after incubation of PRP from healthy donors with glucose, mannitol and Tyrode’s buffer (control) on a wheel agitator for 4 days at 37 °C. More experimental details are given in the Materials and Methods section. The asterisk denotes the particular significances estimated with ANOVA with repeated measurements, followed by post hoc Dunnet’s test: in vitro platelet adhesion: * *p* < 0.001, control < mannitol = glucose; CD61 expression: *p* < 0.001, control < mannitol = glucose.

As hyperglycemia is the most pronounced pathological alteration associated with diabetes and as we have shown significant glucose adducts to GPIIb/IIIa, it was examined whether hyperglycemia per se can modulate blood platelet adhesion. Four-day exposure of platelets to glucose or mannitol led to a significant decrease in adhesion under flow conditions (Figure 5A). The expression of GPIIIa on platelets incubated with glucose or mannitol was lower than that on platelets incubated in normoglycemic conditions (Figure 5B). Plasma-soluble GPIIIa did not differ among the groups (Figure 5C). The glucose and mannitol samples were characterized by higher numbers of GPIIIa-positive particles in incubates but these differences were insignificant, unlike those observed in patients (supplementary Table S1).

### 2.4. Simple Associations between Selected Parameters of Platelet Function and Markers of Glycemic Control

Since hyperglycemia in vitro had a pronounced effect on platelet GPIIIa expression, association analysis was performed to verify whether markers of glycemic control in our study group correlated with GPIIIa expression or its soluble fraction in plasma. The number of platelet microparticles in plasma was found to negatively correlate with the expression of GPIIIa on blood platelets and positively correlate with those of plasma-soluble GPIIIa; this was observed both when combined diabetic and non-diabetic groups were analyzed (R*_S_* = −0.208, *p* = 0.016 and R*_S_* = 0.342, *p* < 0.001, respectively) and for the diabetic group alone (R*_S_* = −0.243, *p* = 0.012, and R*_S_* = 0.419, *p* < 0.001, respectively). In turn, no significant association was observed between soluble GPIIIa in plasma and the expression of GPIIIa on platelets. Interestingly, the concentration of soluble GPIIIa in plasma of diabetic patients correlated positively with fasting glycemia (R*_S_* = 0.279, *p* = 0.004). The amount of platelet microparticles in plasma positively correlated with: i) glycation of platelet membrane proteins (R*_S_* = 0.287, *p* = 0.003 in combined diabetic and non-diabetic groups and R*_S_* = 0.243, *p* = 0.012 in diabetic group); ii) fasting glycemia (R*_S_* = 0.234, *p* = 0.015 in combined diabetic and non-diabetic groups and R*_S_* = 0.285, *p* = 0.03 in diabetic group)**;** and to a lesser extent with iii) HbA_1c_ (R*_S_* = 0.175, *p* = 0.071 in combined diabetic and non-diabetic groups, R*_S_* = 0.183, *p* = 0.059 in diabetic group).

### 2.5. Predictors of Platelet Functioning in Non-diabetic and Type 2 Diabetic Patients: Multivariate Analyses

The purpose of this part of the analysis was to determine which sets of variables and which variables within these sets, predict the non-diabetic and type 2 diabetic patients with the greatest accuracy. Several different multivariate approaches were employed to better characterize the discriminations between non-diabetic and type 2 diabetic patients based on the variables describing platelet function and non-enzymatic glycosylation of proteins: logistic regression (LR), linear discriminant analysis (DA), canonical analysis (CA), ROC analysis (ROC) and a few data mining (DM) techniques (MAR Splines regression, support vectors, naïve Bayes and K-nearest neighbours).

The variables were assigned to two subgroups. The first comprised platelet function variables: flow cytometry markers of resting and ADP or TRAP-agonized whole blood platelets, *p*-selectin and the activated GPIIb/IIIa complex, plasma-soluble P-selectin and GPIIIa, platelet adhesion to fibrinogen or vWF, fibrinogen binding to platelets, small and large platelet microparticles. The second comprised the markers of impaired carbohydrate metabolism: plasma fasting glycaemia, plasma fructosamine, glycated haemoglobin and glycation of platelet proteins.

The contributions of particular variables were always assessed upon adjustment for sex and age. In general, amongst variables characterizing protein glycation, the most significant predictors of diabetic state were platelet protein glycation (LR, ROC, DA, CA,DM), glycated haemoglobin (LR, ROC, DA, DM) and fructosamine (LR, ROC, CA), while a lower ‘power’ to discriminate diabetics and non-diabetics was ascribed to the variables describing platelet function: Fg binding to platelets (LR, ROC, DA, CA, DM), plasma-soluble GPIIIa (LR, ROC, DA, CA, DM), platelet surface membrane GPIIIa abundance (LR, ROC, DA), platelet microparticles (DA), the expression of the activated GPIIb/IIIa complex in ADP-activated cells (DA, ROC, CA, DM) and plasma-soluble P-selectin (LR, ROC, CA, DM) (for more details see Supplementary Materials*,* Table S2).

## 3. Discussion

### 3.1. Characterization of Activation, Reactivity and Protein Glycation in Platelets from Diabetic Patients

We hypothesized that blood platelets from diabetic humans interact with fibrinogen and/or vWF under flow conditions to a higher extent than those from their non-diabetic counterparts. To verify this hypothesis, we used blood from diabetic patients (T2DM) and from sex- and age-matched non-diabetic volunteers. First, the glycation status of the platelets in our studied groups was characterized. Platelet proteins of T2DM patients were glycated to a higher extent, indicating that biochemical modification occurs despite the relatively short life span of platelets in circulation. These results are in agreement with previously published studies which show enhanced glycation of platelet membrane proteins in patients with diabetes [8,9].

Since GPIIb/IIIa is the most abundant protein in platelet membranes [10], which makes it the most likely target for glycation, and because of its key role in the interaction between platelets and activated endothelium, we also performed LC-MS/MS analysis to identify specific glycation sites in the protein. This method did not allow for quantitative comparison of glycation extent between diabetic and non-diabetic individuals. High variability in terms of detected glycation sites in both groups indicated that there were no privileged sites for glycation. The question arises as to whether the identified sites of glycation could affect the function of the protein. There are several sites in the primary structure of GPIIb/IIIa identified as crucial for ligand binding: region 294-314 [11], D224 [12], Y313-L332, G265-Q284, P57-Q64 [13] in GPIIb, and S211-G221 [14], residues 349-422 [15], G184-G193 [16] in GPIIIa. The region spanning from E749 to N756 in GPIIIa has been shown to be crucial for stabilizing the integrin–ligand interaction [17]. None of the lysines identified as glycation sites in our studies were located in the aforementioned regions. Based on these data, it is unlikely to assume that glycation of GPIIb/IIIa may directly affect ligand binding. At the same time, it has to be taken into account that the total number of glycated lysines detected by LC-MS/MS—12 per GPIIb/IIIa—is still below the number of ^14^C-glucose molecules which were found to incorporate to GPIIb/IIIa in our studies, i.e., 18 moles of glucose per mole of GPIIb/IIIa, in conditions roughly corresponding to that existing in vivo. This observation, along with the fact that LC-MS/MS analysis did not manage to achieve full coverage of the GPIIb/IIIa molecule, implies that some sites of glycation might still have not been identified in our studies.

Since basal activation and reactivity of platelets may affect their adhesion potential, these parameters were assessed by flow cytometry. Platelets from diabetic patients were characterized by a slightly higher expression of P-selectin and that of an active form of GPIIb/IIIa. In response to low concentrations of agonists, platelets were either not responding or, if they responded, diabetic platelets were activated slightly more so than non-diabetic ones. In turn, in higher doses of agonists, platelets from both groups either responded similarly or diabetic platelets responded to a lesser extent than non-diabetic platelets. This pattern of opposite platelet response to low and high doses of agonists has been previously reported in our study in diabetic patients, and in mice models of diabetes [18,19]. Interestingly, the population of smaller platelets was elevated in diabetic patients. The presence of a large number of smaller platelets usually means their exhaustion due to less or more frequent episodes of activation in the bloodstream. Moreover, the fraction of platelet aggregates was increased in patients with diabetes. These results confirm our observation of increased priming of diabetic platelets, as revealed by the elevated surface expression of P-selectin and the activated form of GPIIb/IIIa on resting platelets. In contrast, the population of larger platelets was smaller in diabetic patients, which may confirm platelet exhaustion or may indicate a reduced rate of thrombopoiesis under hyperglycaemic conditions. In our study, patients with T2DM were characterized by high fasting glycaemia 9.5 ± 2.7 mM (mean ± SD), high fraction of HbA_1c_ (on average 83 mmol/mol (9.7%) ± 3 mmol/mol (2.4%); mean ± SD), and elevated fructosamine levels (485 ± 250 µM/mg protein; mean ± SD). The non-diabetic donors had fasting glycaemia and HbA_1c_ in the normal range (3.9–5.5 mM for glucose; <37 mmol/mol (5.5%) for HbA_1c_). These data indicate that the diabetic patients in our studied group had poor metabolically controlled DM with a higher glucose concentration independently in the treatment with antihyperglycemic drugs (insulin in various regiments, metformin, sulphonylurea drugs). It was shown that poor glycemic control in patients with type 2 diabetes mellitus was associated with higher levels of urinary concentration of thromboxane metabolite [20], and with increased platelet aggregation [21]. This was further supported by the findings of the positive correlation between HbA_1c_ and MPV [22], suggesting that diabetes favors formation of ‘fresh’, more reactive platelets. Contrary to those studies, in our study, lower MPV was observed in T2DM patients compared to control.

It can be summarized that platelets from diabetic patients are characterized by a lower threshold of activation and are more ‘exhausted’, which is revealed by a lower response to strong stimuli. Although a general notion exists that platelets from diabetic patients have higher basal activation and reactivity, differences occur between reports regarding the extent of these alterations. For example Israels et al. noted that percentage of P-selectin-positive and PAC-1 binding platelets were increased in diabetic patients to about 2% from about 1% in control group [23]; the reactivity of blood platelets in patients with relatively well-controlled DM (HbA_1c_ = 50 mmol/mol (6.7%)) was shown not to differ from that measured in healthy individuals [24]; Soma et al. reported increased basal activation status in DM patients but these results are difficult to interpret as the basal expression of P-selectin in the control group reached 71%, which is a rather unexpected value for non-activated platelets [25].

Notably, using several types of multivariate statistical analysis, we identified which of the analyzed variables characterizing impaired glucose metabolism on one hand, and platelet function on the other, contributed most to the significant discrimination of non-diabetic individuals and type 2 diabetic patients. As expected, the variables describing the extent of protein glycation, despite their poor statistical tolerance, constituted the top ranked candidates. The contributions of platelet function hallmarks were much weaker; however, all the used techniques, i.e., discriminant and canonical analyses, logistic regression or data mining tools, agreeably pointed to the same factors: the abundance of GPIIIa in surface membranes, Fg binding to platelets, plasma-soluble platelet membrane glycoproteins, the expression of the activated GPIIb/IIIa complex upon low ADP agonization and platelet microparticles.

An intriguing discrepancy was observed between the expression of P-selectin on platelets and the concentration of soluble P-selectin in plasma. Compared to controls, platelets from diabetic patients had a higher expression of P-selectin, while the concentrations of soluble P-selectin in plasma was lower in T2DM patients. This pattern is very likely to result from very recent activation episodes of circulating platelets. Such episodes, however, must have been so occasional that platelets in diabetic patients did not become exhausted and remained hyperreactive towards lower concentrations of ADP and TRAP. However, the borderline discrimination with respect to the membrane expression of P-selectin could indicate that the activation episodes were probably too weak to trigger the enhanced release of P-selectin from intraplatelet α-granules. Lastly, it may be also hypothesized that P-selectin very likely binds to the inflamed vascular wall of T2DM patients to a higher extent, which effectively decreases its concentration in plasma. Interestingly, a lower number of P-selectin-positive microvesicles has been previously observed in patients after stroke without recurrent vascular event during the observation period [26]. This observation was also explained by the possible platelet exhaustion.

### 3.2. Evaluation of Adhesion Potential of Platelets from Diabetic Patients

The blood platelets from diabetic patients were not found to demonstrate enhanced adhesion in either of the two applied protocols. Importantly, the platelets in our studied group of diabetic patients were both functionally and structurally altered, as shown by the results of our cytometric studies and assessment of membrane protein glycation. It should be stressed that neither the observed alterations of platelet activation status nor their reactivity unambiguously suggest that enhanced adhesion of platelets in DM group is an expected outcome. The increase in the percentage of platelets with the active form of GPIIb/IIIa was rather minor: from 4% in the non-diabetic participants to 8% in the diabetic participants. Therefore, DM patients demonstrated approximately only 4% higher platelet population with the active form of the receptor compared to controls. Although the increase in reactivity observed only for some agonists was significant, it was also low. Apparently, such a low increase in platelet responsiveness does not result in a significant increase in adhesion.

To our knowledge, these studies were the first to evaluate firm platelet adhesion from diabetic patients under flow conditions to fibrinogen and vWF. A previous report on platelets adhesion under flow conditions to matrices formed by endothelial cells or fibroblasts showed no difference between the adhesion of platelets from diabetic patients and non-diabetic individuals [27]. At the same time, there are conflicting reports on platelet adhesion under flow conditions in vitro in animal models of diabetes. No such increased interactions were observed in our previous in vitro studies in mice with 3-month STZ-induced diabetes [28]. However, it was recently shown that platelets from STZ diabetic mice adhered to a higher extent to fibrinogen in flow conditions in vitro [29]. A possible explanation for this discrepancy may lie in the different criteria applied to define platelets as adherent. In the referenced report, platelets were considered adherent if they were not displaced for at least two seconds or, as stationary platelets, if they made only slight motions for at least ten seconds. Therefore, the criterion included relatively short events of immobilization. In our studies, only these platelets which remained adherent throughout washing, fixation and labelling steps were counted. The referenced studies therefore provided evidence that blood platelets in diabetic conditions acquire the capability to interact more readily with the substrate. We suggest that this increased ability of interactions may not necessarily lead to formation of firm adhesions. At the same time it has to be emphasized that short-lasting, transient interactions of platelets with the vascular wall are possibly of high importance in the process of formation of atherosclerotic plaques as they lead to the deposition of platelet-derived cytokines on the vascular wall [30].

Thus, based on our results, we can conclude that we found no evidence that platelets from diabetic patients demonstrate any enhanced ability to form firm adhesions under flow conditions to fibrinogen and vWF under shear rates similar to those existing in a normal artery. It is important to emphasize that the aim of our study was to understand how platelets can contribute to the early stages of atherosclerosis. For this reason, the experimental conditions were designed to model platelet interactions with the walls of vessels not yet affected by atherosclerosis, i.e., the subendothelial matrix proteins are not yet exposed and shear rates are still in the range of a normal artery and not that of a narrowed one.

### 3.3. Expression of GPIIIa on Blood Platelets from Diabetic Patients

More detailed analysis of the flow cytometry data for blood platelet activation revealed that blood platelets in diabetic patients were characterized by the decreased expression of GPIIIa. This effect was accompanied by an increase in soluble GPIIIa concentration in plasma, as assessed by ELISA, and by the number of GPIIIa positive particles smaller than 0.5 µm. The question arises as to whether a decrease in GPIIIa on platelets can be explained by the increased release of GPIIIa-positive PMPs to the plasma. Association analysis revealed that expression of GPIIIa on platelets negatively correlated with the amount of GPIIIa-positive platelet microparticles in plasma, i.e., a decrease in GPIIIa expression on the platelets of a given donor was accompanied by an increase in GPIIIa-positive platelet microparticles in plasma. No such association was found for plasma-soluble GPIIIa assayed by means of ELISA test. These apparently contradicting findings could be explained by assuming that platelets are not the sole source of GPIIIa. The integrin is also expressed on endothelium and the increase in plasma-soluble GPIIIa observed in DM may be in part of endothelial origin. In turn, GPIIIa released from platelets would circulate in the form of microparticles. It has previously been shown that circulating MPs are mostly of platelet or megakaryocyte origin [31].

The decreased GPIIIa number contrasts with the observed increase in the expression of the active form of GPIIb/IIIa in the DM platelets. This apparent contradiction may be explained by the possibility that more GPIIb/IIIa copies assume an active conformation in diabetic conditions, despite fewer of them being present.

Interestingly, in our present study, both the lower abundance of GPIIIa in platelet surface membranes, elevated content of GPIIIa-positive microparticles and higher plasma concentrations of soluble form of GPIIIa represented the topmost significant platelet function markers contributing to the discrimination between non-diabetic and type 2 diabetic individuals. Furthermore, the abovementioned reduced abundance of GPIIIa in surface membranes of platelets from type 2 diabetic patients on the one hand, and the glycation of platelet proteins on the other, appeared to be the most significant contributors to the associations between platelet function and protein modifications by glucose.

Thus, we suggest that diabetes contributes to an enhanced release of GPIIIa from platelets and that in plasma, this integrin exists mostly on the surface of microparticles rather than as free molecules. Elevated levels of platelet-derived microparticles have been reported previously in diabetic individuals [32,33]. However, some contradiction exists in the literature regarding the GPIIIa levels on platelets in diabetes. Previously published studies reported an increase in GPIIb/IIIa exposed on platelets in diabetic patients [34]. In turn, Watala et al. reported that expression of GPIIIa on platelets was increased in juveniles with diabetes, while the total amount of the protein in diabetic platelets was decreased, as measured by flow cytometry [35]. The authors speculate that due to physicochemical alterations in platelet membranes, GPIIIa is exposed to a higher extent on the membrane, which favors its release and subsequent renewal of its pool from internal stores. This process eventually leads to a decrease in total GPIIIa in platelets. It may be suggested that depending on the studied cohort, this ability of platelets to restore the pool of the integrins is worsened to such an extent that platelets finally present lower amount of copies of the protein.

It is known that diabetic patients are characterized by a prothrombotic state [36]. A decreased number of GPIIIa on platelets from diabetic patients apparently does not impair the ability of platelets to form thrombus which can be explained by the overall high number of GPIIb/IIIa copies per cell. At the same time, GPIIIa-bearing PMPs of which the levels are elevated in diabetic patients constitute a prothrombotic surface and are considered as one of the important factors of the procoagulable state in this disease [37].

### 3.4. In Vitro Effects of Hyperglycemia on Blood Platelets’ Adhesion Potential and GPIIIa Expression

Interestingly, in our studies the amount of platelet microparticles in plasma positively correlated with some of the parameters of glycemic control, as well as with glycation of platelet membrane proteins. This confirmed the notion published previously that hyperglycemia accounts for the process of platelet microparticles formation. We aimed to reproduce the observed effect in in vitro conditions by exposing platelets from healthy individuals to glucose concentrations seen in DM patients. We observed lower adhesion and decreased expression of GPIIIa on platelets as an effect of both glucose and mannitol, which suggests that hyperglycemia per se is a factor which decreases the adhesion potential of platelets, rather than increasing it. Since the same effect was observed for platelets incubated with mannitol, the alterations should be attributed to the osmotic effects of glucose. Surprisingly, the decrease in GPIIIa level on platelets was not associated with any significant increase in the soluble form of this antigen. This can attributed to either better preservation of the antigen or to a substantial contribution of endothelial-derived GPIIIa in the intravital conditions.

Our observation that hyperglycemia in vitro affects integrin expression on platelets from normoglycemic donors is in accordance with other reports. It has been shown that acute, short-term hyperglycemia in vitro resulted in a decrease in GPIIb expression on platelets [38]. Acute hyperglycemia in patients with type 2 DM increased shear stress-induced platelet activation, suggesting that relatively short exposition to high glucose concentration in vivo can also have a detrimental effect on platelets [39]. The mechanism by which hyperglycemia exerts these effects may to a large extent be based on osmolarity. In our studies, the effects of high glucose concentration were reproduced by equimolar concentration of mannitol. It has hence been reported that 60-min exposure of platelets from non-diabetic donors to a high concentration of glucose and an equimolar concentration of mannitol resulted in an enhanced activation and reactivity [40].

Although hyperglycemia is the most pronounced biochemical alteration which affects blood platelets in diabetes, it is possible that higher concentrations of triglycerides, lipoproteins or certain cytokines can also play a role. It cannot be excluded that the effects of these factors when tested in vitro could have an opposite effect on platelet adhesion than hyperglycemia. This remains an open question and a subject for further studies.

### 3.5. Adhesion Potential of Platelets In Vitro and In Vivo and Its Role in Atherosclerosis Development

No evidence was observed for an enhanced firm adhesion of blood platelets from diabetic patients under flow conditions in vitro; this result may seem to contradict previous in vivo studies in animal models of diabetes. One possible explanation for this discrepancy is that such enhanced interactions are not dependent on platelet function in the first place. Instead, the main compounding factor may be the functional condition of vascular endothelium. In other words, increased basal activation status and hyperreactivity of blood platelets to the extent observed in diabetes do not seem to be a prerequisite for an increased frequency of interactions with the vascular wall in vivo. Decreased expression of GPIIIa on diabetic platelets may additionally contribute to the lack of enhanced firm adhesion of ‘diabetic’ platelets in vitro.

Although the contribution of platelets to the development of atherosclerotic lesions is undisputed, the exact mechanism of this contribution is not clear. There are studies which have suggested that an increased potential to firm adhesion of platelets is not mandatory for this process [30,41]. Our results support this notion. Therefore, our failure to show enhanced ability of platelets to form firm interactions with fibrinogen and vWF under flow conditions does not undermine the concept of the role of platelets in atherosclerosis development.

### 3.6. Study Limitations

The adhesion potential of platelets was determined by measuring their adhesion to fibrinogen and von Willebrand factor. Although these proteins are among the most often studied substrates in adhesion assays, they do not represent the entire set of factors influencing platelet adhesion to the inflamed endothelium. Other proteins which take part in this process include P-selectin and junctional adhesion molecule-A.

Diabetic patients and non-diabetic individuals enrolled to the study constituted a heterogeneous group with respect to metformin and statins usage. There are numerous reports that these substances decrease platelet activity. Therefore, this heterogeneity may potentially influence the outcomes of our studies. To minimize this effect, we attempted to balance the ratio of statin-treated individuals between studied groups: eventually, 60% of the patients in the diabetic group and 41% in the non-diabetic group were taking statins. For the obvious reasons, it was not possible to eliminate metformin-treated diabetic patients or to introduce metformin-treated individuals to the control group. However, only 50% of diabetic patients were treated with metformin. It is important to stress that despite these confounding factors, blood platelets of diabetic patients presented increased basal activation status and reactivity in response to TRAP.

## 4. Materials and Methods

### 4.1. Chemicals

Fluorolabelled monoclonal mouse anti-human antibodies: anti-GPIIIa/PerCP (BD Biosciences cat no. 340506), anti-GPIIIa/PE (BD Pharmingen™ cat no. 555754), anti-P-selectin/PE (BD Biosciences cat no. 348107), PAC-1/FITC (anti-activated GPIIb/IIIa complex) (BD Biosciences cat no. 340507), CellFIX were purchased from Becton Dickinson (San Diego, CA, USA). Oregon Green 488-labelled human fibrinogen was from Molecular Probes (Eugene, OR, USA). Bovine serum albumin fraction, Arg—Gly—Asp—Ser (RGDS) peptide, Ser-Phe-Leu-Leu-Arg-Asn-Pro-Asn-Asp-Lys-Tyr-Glu-Pro-Phe (TRAP, thrombin receptor-activating peptide) peptide, adenosine 5′-diphosphate sodium salt (ADP) and other chemicals were from Sigma (St. Louis, MO, USA), unless otherwise stated. Isolated GPIIb/IIIa was purchased from Calbiochem (Darmstadt, Germany). Sepharose 2B gel was from Amersham Pharmacia Biotech (Uppsala, Sweden). Soluble P-selectin and GPIIIa were assayed with the use of ELISA tests from Cusabio (Wuhan, China). Glycated serum protein assay (GlycoCap) was purchased from Diazyme Laboratories (Poway, CA, USA). Fibrinogen from human plasma (containing ≥90% clottable proteins) and D-phenylalanyl-prolyl-arginyl chloromethyl ketone (PPACK) were from Calbiochem (Darmstadt, Germany). Human von Willebrand factor was purchased from Abcam (Cambridge, MA, USA). Water for solution preparation and glassware washing was passed through an Easy Pure UF water purification unit (Thermolyne Barnstead, Dubuque, IA, USA).

### 4.2. Blood Donors and T2DM Patients Recruitment–Exclusion Criteria

The study enrolled 61 patients (31 men, 30 women; mean age 56.8 ± 8.2 years; min–max age 40–65 years) with type 2 diabetes mellitus (T2DM), diagnosed based on the current guidelines of European Association for the Study of Diabetes (EASD) criteria, and 57 donors as a control group (without DM; 29 men and 28 women; mean age 52.9 ± 9.7 years; min–max age 36–65 years), who gave signed written consent for participation in the study. The exclusion criteria for both groups were as follows: patients under 18 years of age, recognized pre-diabetes condition, pregnancy, acute infection, chronic inflammatory disease including autoimmune disturbance, current cancer, current or previous blood and bone marrow cancer, liver and spleen disease, arterial or venous thromboembolic events within 6 months, surgical procedures in the last 6 months, current anticoagulant therapy and use of antiplatelet drugs. A precise medical history of co-morbidities and medication was collected for each individual enrolled in the study. Physical examination and anthropometric measurements (for body mass index (BMI) assessment) were conducted for all participants. For every participant, venous blood was drawn to measure complete blood count and mean platelet volume (MPV), serum fasting glucose, glycated haemoglobin (HbA_1c_), fructosamine level, as well as to evaluate renal function (creatinine level and glomerular filtration rate (GFR), using CKD-EPI formula), liver enzyme levels (alanine aminotransferase (ALT) and aspartate aminotransferase (AST)), a fasting lipid profile (including total cholesterol, high-density lipoproteins, low-density lipoproteins (LDL) and triglycerides) and to perform platelet functionality assays. The level of HbA_1c_ was evaluated with immunoturbidimetric assay (ITA, Beckman Coulter, CA, USA). The clinical characteristics of T2DM patients and control donors are shown in Table 2.

The median duration of diabetes was 2.5 years (from less than 1 year to 18 years*,* interquartile range of 1–6 years). Only 5 patients had HbA_1c_ level below 53 mmol/mol (7%) (mean HbA_1c_ 83 mmol/mol (9.7%) ± 3 mmol/mol (2.4%)). Nineteen patients with T2DM did not use any hyperglycaemic agents (13 patients with newly diagnosed disease and six, who had not been treated despite the occurrence of diabetes lasting for over a year). The remaining diabetic patients had been treated with various antihyperglycemic drugs: nine with insulin monotherapy, nine with a combination of insulin and metformin (one patient used additionally glimepiride), 22 with metformin (12 as monotherapy, eight with sulfonylurea and two with sodium-glucose co-transporter 2 inhibitors) and two treated only with sulfonylurea. Twenty-two patients were diagnosed with chronic diabetes complications: 17 (28%) demonstrated diabetic polyneuropathy, 13 (21%) demonstrated diabetic retinopathy, 10 (16%) presented with diabetic kidney disease and three (5%) had diabetic foot syndrome. In comparison to people with DM, the control group had significantly lower BMI, as well as lower fasting glucose, fructosamine and triglycerides concentrations, but higher MPV values. Thirty-two T2DM patients and 11 control subjects were treated with statins, additionally 5 T2DM patients and 1 control subjects used fibrates.

Blood for the in vitro experiments (the incubation with glucose or mannitol) was obtained from 16 healthy volunteers (10 women and six men) with a mean age of 28.9 ± 10.6 years. All the enrolled donors were non-smokers, and none had taken aspirin or other drugs affecting platelet function for at least 10 days prior to blood collection or had a history suggestive of underlying haemostatic disorders. The concentrations of glucose in the plasma samples from healthy volunteers were in the reference range (4.1–5.5 mM).

The study was performed under the guidelines of the Helsinki Declaration for human research and approved by the Medical University of Lodz committee on the Ethics of Research in Human Experimentation (approval number: RNN/122/15/KB, approval date:17 February 2015).

### 4.3. Blood Collection and Preparation for Ex Vivo and In Vitro Analysis

Blood was collected into a vacuum tube containing 0.105 M buffered sodium citrate and further processes to obtain platelet-rich plasma (PRP) or platelet poor plasma (PPP). Isolated platelets were prepared by gel filtration as described earlier [42]. Details of the procedure are given in Supplementary Materials.

Blood was withdrawn with special care to avoid undesirable activation of platelets. All measurements of platelet reactivity were performed within two hours after blood withdrawal.

### 4.4. Evaluation of Blood Platelet Adhesion

In the first set of experiments, platelet adhesion was tested in whole blood samples. Samples were normalized for platelet count. For this purpose, whole blood was diluted with donor’s autologous platelet poor plasma (PPP) to obtain a platelet count of 100,000 plt/µL. Dilutions were made on the basis of platelet count estimated by a hematology analyzer (Sysmex XS-800i, Norderstedt, Germany) and performed after blood collection. Hematocrit values of such diluted blood varied between 7% and 30%. Blood was supplemented with the thrombin inhibitor PPACK at a final concentration of 62.5 µM and with PGE_1_ at a final concentration of 2 µg/mL. Prostaglandin E_1_ was added to decrease blood platelet aggregation which occurs in its absence (Figure S5). 

In the second set of experiments, suspensions of blood platelets combined with RBCs were prepared. Blood platelets were isolated as described in the Blood Collection and Preparation for Ex Vivo and In Vitro Analysis section. Autologous RBCs were prepared by triple washing with PBS without calcium and magnesium. RBC and isolated platelets were suspended in Tyrode’s solution (composition is given in the Supplementary Materials), to obtain the platelet count of 100,000 platelets/µL and haematocrit of 40% and 50%.

For the third series of experiments, PRP was prepared as described in the Incubation of Platelets with Glucose or Mannitol section (Supplementary Materials).

The experiments were performed with the use of VenaFlux platform (Cellix, Ireland). Channels of Vena8 Fluo+ biochip were coated with human fibrinogen (200 µg/mL) or human von Willebrand factor (20 µg/mL) overnight at 4 °C and blocked with BSA (1 mg/mL) for one hour at 4 °C. Biochip was mounted on a thermally controlled stage of inverted AxioVert microscope (Zeiss, Germany) assuring constant temperature of 37 °C throughout the experiment. Prior to measurements, the channels were washed with PBS for 2 min at 2 dynes/cm^2^. Blood samples or platelet suspension prepared as described above were recalcified by addition of CaCl_2_ to a final concentration of 1 mM and passed for 1 min through microchannels at a shear force equal to 20 dynes/cm^2^. Perfusion time was chosen to assess platelet adhesion to the protein-coated surface and to avoid platelet–platelet aggregate formation. Afterwards, the channels were washed with CellFIX solution (5 dynes/cm^2^, 2 min) to remove non-adherent platelets and incubated for 1 h at RT to fix the adherent cells prior to labelling with antibodies. Firmly adherent blood platelets were stained for 30 min with PE-conjugated anti-GPIIIa antibodies. Visualization was performed with the use of fluorescence microscope and imaged in four different fields along the channel. The images were taken at sites approximately 20%, 40%, 60% and 80% of the length of the channel (28 mm). Each image was 200 µm × 200 µm in size. The four images therefore spanned approx. 1/30 of its entire length. Afterwards, either a number of platelets or the area covered by platelets were assayed with the use of ZEN software (Zeiss, Germany).

### 4.5. Measurement of Platelet Activation and Reactivity by Flow Cytometry

To analyze platelet activation, the basal expression of the active forms of GPIIb/IIIa, P-selectin, and GPIIIa and platelet ability to bind exogenous fibrinogen were evaluated by flow cytometry (FACSCanto II, Becton Dickinson, San Diego, CA, USA), as described previously [43,44].

The median fluorescence intensity of anti-GPIIIa MoAbs bound to the stained platelets and the median FSC value of GPIIIa-positive cells (blood platelets) were read in every sample in order to evaluate the association between the median sizes of the analyzed blood platelet populations and the GPIIIa content as described previously [5]. For details of the statistical approach see Section 4.10. Statistical Analysis.

### 4.6. Plasma Markers of Platelet Activation (PMP, Plasma GPIIIa, Soluble P-selectin)

The level of platelet-derived microparticles (PMP) was measured in the platelet-free plasma (PFP) samples obtained from both T2DM and control subjects, and from PRP after in vitro incubation with glucose or mannitol (after incubation PRP samples were centrifuged for 12 min, 190× *g* to obtain PPP and platelets). The PFP samples (PPP from citrate blood were centrifuged for 5 min at 10,000 × *g*) were stored at −80 °C until measurement. The PFP samples were thawed directly prior to labeling, diluted twice with a sodium chloride solution (5 µL of PFP and 5 µL of NaCl) and incubated with 2 µL of anti-GPIIIa/PE antibodies. The samples were stained in darkness at RT for 20 min, then diluted with sodium chloride solution (the dilution factor was 62.4) and directly analyzed by flow cytometry. The acquisition time was 100 s with a flow rate of 10 µL/min. The measurement of microparticles was performed on CytoFLEX cytometer (Beckman Coulter Inc., Atlanta, GA, USA), which offers possibilities to measure small particles from 100 nm onward. Details are given in supplementary materials (Figure S1).

The concentration of plasma soluble GPIIIa and P-selectin in platelet-free plasma (PFP) was assessed by dedicated ELISA tests, according to the manufacturer’s instructions. Samples were diluted 2-fold for detection of plasma soluble P-selectino fit within the standard curves range. No dilution of plasma was necessary for determination of plasma-soluble GPIIIa.

### 4.7. Assessment of Glycation Status of Platelet Membrane Proteins from Patients with the Use of Borohydride-[^3^H]

Platelets were suspended in PBS and supplemented with 1 mM PMSF, 1 mM DTT and 1 mM EDTA. Cell lysis was performed by five cycles of Yeda press disintegration (Yeda Scientific Instruments*,* Rehovot, Israel). Subsequently, samples were centrifuged at 100,000 × *g*, 1 h, 4 °C. To remove most of peripheral proteins bound to membrane fragments, the pellet was washed in 100 mM sodium carbonate buffer, pH 11.3 and centrifuged again at 100,000× *g*, 1 h, 4 °C to sediment membrane fragments. The resulting pellet was suspended in PBS with 2% SDS and used for membrane protein glycation measurement. Glycation was determined by a reduction with sodium borohydride-[^3^H] according to Watala et al. [45]. Mass spectroscopy was performed on glycated GPIIb/IIIa (supplementary data).

### 4.8. In Vitro Glycation of GPIIb/IIIa with ^14^C-glucose

GPIIb/IIIa (0.5 mg/mL) was incubated with 30 mM glucose and 3 µM [^14^C]-glucose (ratio 1:10,000, radioactive:non-radioactive glucose) for 5 days at 37 °C. The detailed procedure of measurement of ^14^C-glucose binding to GPIIb/IIIa is given in Supplementary Materials.

### 4.9. Assessment of Glycation of Plasma Proteins with the Use of Glycated Serum Protein Assay

Glycated serum protein assay was done according to manufacturer’s instructions (Diazyme Laboratories, Poway, CA, USA). The fructosamine level was standardized on protein concentration determined by BCA assay (Bicinchoninic Acid Kit, Sigma Aldrich, St. Louis, MO, USA).

### 4.10. Statistical Analysis

Data were expressed as mean ± SD or median and IQR (interquartile range: lower (25%) to upper quartile (75%)), depending on data distribution and variance homogeneity (the Shapiro–Wilk test and Brown–Forsythe test, respectively) and the scale of data (continuous vs. categorical). The following parametric/nonparametric analyses were used for either raw or Box-Cox transformed data: Student’s t-test/Mann–Whitney U test, general linear models (various models of ANOVA and post-hoc multiple comparisons tests, ANCOVA in data adjustment for confounders) as inference testing methods, standard/partial Pearson’s linear/Spearman correlation for associations, logistic regression, discrimination analysis, canonical analysis and data mining as multivariate techniques (for more details see Supplementary Materials). Due to the relatively small sample sizes and the low statistical power of the estimated inferences in the majority of calculations, the resampling bootstrap technique (1000–10,000 iterations) was used to determine the likelihood of obtaining the revealed differences due to pure chance; in such circumstances, we refer to the bootstrap-boosted test statistics instead of the classical approach. For some variables, in order to better characterize the heterogeneity in platelet size and in the abundance of GPIIIa in surface platelet membranes in platelets from control and diabetic subjects, we employed the generalized expectation-maximization (EM) and k-means cluster analysis (STATISTICA Data Miner) (for more details see Supplementary Materials). Statistica v. 13.1 (Dell Inc., Tulsa, OH, USA), StatsDirect v.3.0.182 (Merseyside (Birkenhead), UK), GraphPad Prism v.5 (San Diego, CA, USA) and Resampling Stats Add-in for Excel v.4 (Arlington, VA, USA) were used in statistical calculations.

## Figures and Tables

**Table 1:** GPIIIa-positive small and large microparticles in blood of non-diabetic individuals and type 2 diabetic patients.

	Non-Diabetic (*n* = 25)	Type 2 Diabetic (*n* = 29)
all microparticles (µL^−1^) small microparticles < 0.5 µm (µL^−1^)	7786 (5092; 23,129) 7662 (4733; 21,821)	17466 (6004; 26,446) * 17937 (5758; 27,076) ^#^
large microparticles 0.5–1 µm (µL^−1^)	545 (328; 703)	416 (273; 608)

Variables, adjusted for age and sex, are presented as medians with interquartile ranges (Q1; Q3). The adjustment was performed on the Box-Cox-transformed with the use of covariance analysis (ANCOVA). The significance estimated with the bootstrap-boosted ANOVA: * *P*_1α_ < 0.035; ^#^ *P*_1α_ < 0.030.

**Table 2:** Characteristics of T2DM patients and control subjects.

Variables	Non-Diabetic Subjects (Controls)	Type 2 Diabetic Patients (T2DM)
BMI (kg/m^2^)	24.5 (22.6; 27.7)	31.5 (27.1; 36.4) ^†††^
Hematocrit (%)	39.8 ± 7.3	43.4 ± 4.8
Erythrocyte count (×10^6^/µL)	4.8 ± 1.0	4.6 ± 0.5
Leucocyte count (×10^3^/µL)	6.8 ± 2.3	7.3 ± 2.7
Platelet count (×10^3^/µL)	244 ± 72	234 ± 69
MPV (fL)	9.7 ± 1.2	8.8 ± 0.9 ^#^
Fasting glucose (mmol/L)	5.45 (4.97; 5.64)	9.3 (6.99; 10.79) ^†††^
HbA_1c_ (mmol/mol)	37 (33; 40)	88 (60; 106) ^†††^
Fructosamine (µmol/mg protein)	295 ± 125	576 ± 316 ^†††^
Creatinine (μmol/L)	72.0 (64.0; 94.4)	81.4 (66.0; 95.8)
GFR (ml/min/1.73 m^2^)	95 (70; 101)	82 (65; 95)
ALT (U/L)	23.0 (19.5; 39.6)	33.9 (20.0; 62.9)
AST (U/L)	57.8 ± 107.7	39.1 ± 29.4
Total cholesterol (mmol/L)	5.01 (3.66; 6.17)	4.64 (3.77; 6.10)
LDL cholesterol (mmol/L)	3.11 ± 1.07	2.99 ± 1.48
HDL cholesterol (mmol/L)	0.98 (0.82; 1.39)	1.10 (0.77; 1.34)
Triglycerides (mmol/L)	1.44 (0.90; 2.02)	2.28 (1.49; 3.17) *
Uric acid (µmol/L)	349 (230; 456)	333 (280; 428)

Variables, adjusted for age and sex, are presented as means ± SD or medians with interquartile ranges (Q1; Q3). Comparisons between the groups were performed on the Box-Cox-transformed adjusted values with the use of the covariance analysis (ANCOVA): * *p* ≤ 0.05; ^#^ *p* < 0.01; ^†††^ *p* << 0.0001. Abbreviations used: BMI, body mass index; MPV, mean platelet volume; HbA_1c_, glycated hemoglobin; GFR, glomerular filtration rate; ALT, alanine aminotransferase; AST, aspartate aminotransferase.

## Supplementary Materials

Supplementary materials can be found at https://www.mdpi.com/1422-0067/21/9/3222/s1. Figure S1. Exemplary dot plots of platelet derived microparticles (PMP) recorded using flow cytometry. Figure S2. Areas of adhered platelets. Areas of individual blood platelets adhered according to the first protocol (whole blood diluted with autologous plasma). Each dot represents median area of platelet assessed in a given blood donor. Lines represent median with IQR. Figure S3. Platelets’ area distribution. Histograms represent distributions of platelets’ areas. Data were cumulated from all samples in a given group (control or DM) run over an indicated protein. Figure S4. Exemplary images of the pattern of platelet adhesion in presence of 50% autologous RBC. Figure S5. Exemplary images of the pattern of platelet adhesion in absence of PGE1. Table S1. Effects of in vitro incubation of platelet-reach plasma with glucose or mannitol on the fractions of GPIIIa-positive microparticles. Table S2. Canonical correlations between the set of blood platelet reactivity parameters and the sets of protein glycation parameters.
