# Supplementary material for: Diabetes and Hyperglycemia Affect Platelet GPIIIa Expression: Effects on Adhesion Potential of Blood Platelets from Diabetic Patients under In Vitro Flow Conditions
Source: Int J Mol Sci. 2020 May 2;21(9):3222. doi: 10.3390/ijms21093222 (PMC7247361; doi:10.3390/ijms21093222)
Supplement: Supplementary file 1 [file ijms-21-03222-s001.pdf]

International Journal of *Molecular Sciences*

Supplementary Materials

Diabetes and Hyperglycemia Affect Platelet GPIIIa Expression. Effects on Adhesion Potential of Blood Platelets from Diabetic Patients under In Vitro Flow Conditions

Tomasz Przygodzki [1,*,†], Boguslawa Luzak [1,†], Hassan Kassassir [1], Ewelina Mnich [1], Magdalena Boncler [1], Karolina Siewiera [1,2], Marcin Kosmalski [3], Jacek Szymanski [4] and Cezary Watala [1]

1 Department of Haemostasis and Haemostatic Disorders, Chair of Biomedical Sciences, Medical University of Lodz, Mazowiecka 6/8, 92-235 Lodz, Poland; boguslawa.luzak@umed.lodz.pl (B.L.); hassan.kassassir1@gmail.com (H.K.); mnich.ewelina@gmail.com (E.M.); magdalena.boncler@umed.lodz.pl (M.B.); karolina.siewiera@umed.lodz.pl (K.S.); cezary.watala@umed.lodz.pl (C.W.)
2 Department of Cytobiology and Proteomics, Chair of Biomedical Sciences, Medical University of Lodz, Mazowiecka 6/8, 92-235 Lodz, Poland
3 Department of Clinical Pharmacology, Medical University of Lodz, Kopcinskiego 22, 90-153 Lodz, Poland; marcin.kosmalski@umed.lodz.pl
4 Central Scientific Laboratory, Medical University of Lodz, Mazowiecka 6/8, 92-235 Lodz, Poland; jacek.szymanski@umed.lodz.pl
* Correspondence: tomasz.przygodzki@umed.lodz.pl
† These authors contributed equally to this work.

Materials and Methods

Blood Collection and Preparation for Ex Vivo and In Vitro Analysis

Blood was collected into a vacuum tube containing 0.105 M buffered sodium citrate for platelet adhesion, flow cytometry, measurements of soluble platelet surface membrane glycoproteins (GPIIIa and P-selectin) and for in vitro incubation with glucose. The blood samples were centrifuged for 12 min at 190 × *g* to obtain platelet-rich plasma (PRP) or were centrifuged for 15 min at 2000 × *g* to obtain platelet poor plasma (PPP). To prepare isolated platelets for the assessment of adhesion assays, 0.5 mL of PRP was layered onto the top of BSA-Sepharose 2B gel column prepared as described earlier (6). The isolated platelets were suspended in Tyrode's buffer (134 mM NaCl, 12 mM $NaHCO_3$, 2.9 mM KCl, 0.34 mM Na_2HPO_4, 1 mM $MgCl_2$, 10 mM HEPES, 5 mM glucose, 0.1% BSA, pH 7.4).

To prepare platelets for the membrane protein isolation, six volumes of blood were mixed with one volume of ACD (acid—citrate—dextrose, 98 mM sodium citrate, 71 mM citric acid, 111 mM glucose, pH 4.5). The blood in the presence of 50 ng/mL prostaglandin E_1 (PGE_1) was immediately centrifuged to obtain PRP. The upper two-thirds of the PRP volume was transferred into a separate tube and centrifuged for 12 min at 2000 × *g* to sediment platelets. The platelet pellet was resuspended in nine volumes of Tyrode's buffer supplemented with one volume of anticoagulant (ACD) and washed once with Tyrode's buffer (800× *g*, 15 min). After centrifugation the platelet pellets were stored frozen at −80 °C until processed for the membrane protein isolation.

Measurement of Platelet Activation and Reactivity by Flow Cytometry

To analyse platelet activation, basal expression of the active form of GPIIb/IIIa, P-selectin, GPIIIa and ability for binding of exogenous fibrinogen were evaluated by flow cytometry (FACSCanto II, Becton Dickinson). Blood platelet reactivity was tested in whole blood after 5 min incubation with ADP (1 and 10 μM) or TRAP (1 and 5 μM). Briefly, to analyse the expressions of surface membrane antigens in non-activated or agonist-stimulated platelets, the 10 μl aliquots of blood were diluted in 90 μl PBS (supplemented with 1 mM $MgCl_2$) and immediately transferred to a mixture of staining antibodies (anti-GPIIIa/PerCP, PAC-1/FITC, anti-P-selectin/PE). The labelling was allowed to proceed for 20 min in the dark and the reaction was subsequently stopped by the addition of 1% CellFIX solution. To evaluate fibrinogen binding, the platelets in whole blood or in RBC suspension were incubated with Oregon Green-conjugated fibrinogen at the final concentration of 30 μg/mL for 2.5, 5, 7.5, 10 and 15 min, RT. Next, the 10 μl aliquots of blood or platelets suspension were diluted in 90 μl PBS and immediately transferred into a FACS tube containing anti-GPIIIa/PE antibodies. The labelling was allowed to proceed for 20 min in the dark and the reaction was subsequently stopped by the addition of 1% CellFIX solution. The percent fractions of specific fluorescence-positive platelets were evaluated after subtracting of nonspecific fluorescence in the anti-GPIIIa/PE-labelled samples. As a negative control of the specific fibrinogen binding to GPIIb/IIIa receptor, blocking peptide (RGDS) was used in the final concentration of 1.25 mM (the samples were incubated with RGDS for 5 min before adding of the Oregon Green-conjugated fibrinogen).

In parallel, the median fluorescence intensity of anti-GPIIIa MoAbs bound to the stained platelets and the median FSC value of GPIIIa-positive cells (blood platelets) were read in every sample in order to evaluate the association between the median sizes of the analysed blood platelet populations and the contents of GPIIIa. Briefly, the groups of large (newly-produced, "fresh") or small (exhausted, "old") platelets in the population of single platelets were evaluated according to the size of these cells. In addition, single and aggregated platelets (GPIIIa-positive cells) in resting, non-stimulated blood were differentiated on the basis of platelet size. The diameter of the platelets in blood samples was determined using a standard curve prepared on FSC calibration beads of the following sizes: 0.5, 1, 2–2.4, 3–3.4, 5–5.9, 7–7.9, 8–12.9, 13–17.9 μm. As the use of artificial beads for any size-related calibrations remains imprecise, since beads and platelets display different refractive indices, our intention was not to determine the exact size of platelets in blood samples, but rather to identify the differences between control and diabetic patients with regard to the groups of newly-produced and exhausted platelets, and in the formation of platelet aggregates. The same algorithm of discrimination of overall platelet population into two separate subpopulations was used to evaluate the abundance of GPIIIa copies on platelet surface on the basis of anti-GPIIIa/PE antibodies-derived fluorescence (for details of statistical approach see '*Statistical analysis*') (5).

Plasma Markers of Platelet Activation (PMP, Plasma GPIIIa, Soluble P-selectin)

The level of platelet-derived microparticles (PMP) was measured in the platelet-free plasma (PFP) samples obtained from both T2DM and control subjects, and in PRP after in vitro incubation with glucose or mannitol. Until measurements, the PFP samples (PPP from citrate blood were centrifuged for 5 min at 10,000 × *g*) were stored frozen at −80 °C. The PFP were thawed directly prior to labeling, diluted twice with a sodium chloride solution (5 μl of PFP and 5 μl of NaCl) and incubated with 2 μl of anti-GPIIIa/PE antibodies. The samples were stained in a darkness at RT for 20 min, then diluted with sodium chloride solution (the dilution factor was 62.4) and directly analyzed by flow cytometer. The acquisition time was 100 s with a flow rate of 10 μl/min. The measurement of microparticles was performed on CytoFLEX cytometer (Beckman Coulter Inc., Atlanta, GA, USA), which offers possibilities to measure small particles from 100 nm onward. This instrument gives an opportunity to use the violet laser for side scatter analysis, which improves the resolution on this parameter (VioletSSC) . The flow cytometer was set up by using fluorescent polystyrene beads (Megamix-Plus SSC, BioCytex, Marseille, FRA), which contain beads in a mixture of 0.16 μm, 0.20 μm, 0.24 μm, 0.5 μm and 1 μm beads (Polysciences, Inc. U.S.). CytoFLEX was used in accordance with the manufacturer's instructions. The data were visualized and quantified by using the Kaluza

Analysis 1.5a Software (Beckman Coulter Inc., Atlanta, GA, USA). The analysis of microparticles was conducted in a rectangular gate which was set between the 0.16 μm and 1 μm bead populations and defined as PMP gate (all PMP) (Figure S1A). This gate was divided into two parts. The first from 0.16 μm to 0.5 μm which is called small PMP gate (sPMP) and the second from 0.5 μm to 1 μm which is called large PMP gate (lPMP) (Figure S1B). The data were analysed separately for each gate as a number of GPIIIa/PE positive events (all PMP, lPMP, sPMP) in comparison to the unstained control (Figure S1C). The examples of this type of analysis are presented in three variants of samples for one patient (Figure S1D-1F). The data are presented as a number of GPIIIa/PE-positive events per microliter, and they were obtained by using the formula: number of GPIIIa/PE-positive events (all PMP,lPMP, sPMP) multiplied by the dilution factor.

Concentration of plasma soluble GPIIIa and P-selectin in platelet depleted plasma (PFP) was assessed by dedicated ELISA tests, according to the manufacturer's instructions. Samples were diluted 2-fold for detection of plasma soluble P-selectin—to fit within the standard curves range. No dilution of plasma was necessary for determination of plasma soluble GPIIIa.

Assessment of Glycation Status of Platelet Membrane Proteins from Patients with the Use of borohydride-[^{3}H]

Platelets were suspended in PBS buffer (2 mL) and supplemented with 1 mM PMSF, 1 mM DTT and 1 mM EDTA. Lysis of cell was performed by five cycles of Yeda press disintegration (Yeda Scientific Instruments, Rehovot, Israel). Subsequently, samples were centrifuged at 100,000 × *g*, 1h, 4 °C. To remove most of peripheral proteins bound to membrane fragments, pellet was washed in 100 mM sodium carbonate buffer, pH 11.3 and centrifuged again at 100,000 × *g*, 1 h, 4 °C to sediment membrane fragments. Membrane protein pellet was suspended in PBS with 2% SDS and used for membrane protein glycation measurement. Glycation was determined by a reduction with sodium borohydride-[^{3}H] according to procedure described previously by Watala, et al.(9). Briefly, 100 μg of platelets membrane proteins was treated with 100 μl of 24 mM with sodium borohydride-[^{3}H] (Hartmann Analytic, specific activity—100 mCi/mmol) and incubated at 37 °C for 2h. After the incubation, proteins were precipitated with 10% trichloroacetic acid and centrifuged for 5 min at 10,000 × *g*. The pellet was dissolved in 1 M NaOH and re-precipitated with trichloroacetic acid. This procedure was repeated 3 times until activity less than 200 dpm was detected in the supernatant. Finally, the pellet was dissolved in 100 mM NaOH and radioactivity was measured. Assuming that 1 M of glucose adduct will be reduced by 1 M sodium borohydride-[^{3}H], results were expressed in nmol glucose per mg of protein.

Incubation of Platelets with Glucose or Mannitol

Platelets in PRP were incubated with 30 mM glucose or 30 mM mannitol (both solutions in Tyrode's buffer) for 4 days in 37 °C on a wheel agitator FALC F200 (FALC Instruments, Italy). PRP treated only with Tyrode's buffer was used as the control. To prevent platelet aggregation during incubation, samples were supplemented with $EDTAK_2$ at final concentration of 5 mM. After incubation, the samples of PRP were supplemented with PGE_1 (50 ng/mL) and centrifuged for 12 min at 2000 × *g* to sediment platelets. The platelet pellet was resuspended in Tyrode's buffer to obtain platelet count of 2×10^8 platelets/mL. Next, platelets were combined with autologous RBC (previously washed 2-times with PBS without calcium and magnesium), to obtain the platelet count of 1×10^8 platelets/mL and haematocrit of 40–50%. The platelets in suspension with RBC were analysed in adhesion under flow, in flow cytometry (the measurement of fibrinogen binding), and mean volume of platelets (MPV) was monitored. Additionally, platelet pellets were frozen in -80°C until the determination of protein glycation in platelet lysates. To monitor of plasma protein glycation, the samples of PPP were frozen and stored at −80 °C until measurements. To determine platelet-derived microparticles (PMP) or plasma soluble GPIIIa concentration, PPP samples were centrifuged for 5 min at 10,000 × *g*, and supernatants were stored frozen at −80 °C until assayed.

Assessment of Glycation of Platelet Proteins In Vitro

After incubation of PRP with Tyrode (control), glucose or mannitol, isolated and washed platelets were lysed in CHAPS buffer with additional sonication (10 cycles, each lasting for 10 s, with 5 sec intervals) on ice. In order to prevent proteins proteolysis, lysis buffer was supplemented with the mixture containing selected inhibitors of proteases, according to a manufacturer's specification. The concentration of fructosamine, product of protein glycation, was measured by nitroblue tetrazolium (NBT) assay. Platelets lysates were incubated with 0.75 mM NBT in 100 mM carbonate buffer (pH 10.8) for 60 min at 37 °C and the absorbance was measured at 530 nm. The fructosamine analog, 1-deoxy-1-morpholino-fructose (1-DMF), was used for the standard curve. Additionally, the concentration of protein in plasma was assayed using BCA kit and the fructosamine levels were shown also as μmoles of DMF per mg of total plasma protein.

In Vitro Glycation of GPIIb/IIIa with ^{14}C-glucose

GPIIbIIIa (0.5 mg/mL) was incubated with 30 mM glucose and 3 μM [^{14}C]-glucose (ratio 1:10,000, radioactive:non-radioactive glucose) for 5 days at 37 °C. Glycated proteins were precipitated with 10% TCA. Briefly, TCA was added to the aliquot of sample at the ratio of 1:4 (*v:v*). Sample was incubated for 5 min at 4 °C and centrifuged for 5 min at 20,000 × *g* at 4 °C, supernatant was discarded and pellet containing proteins was washed 5 times with ice-cold PBS. Pellet was dissolved in 1 N NaOH, protein concentration was measured using BCA kit. Sample was added to liquid scintillation cocktail (containing 2,5-Diphenyloxazole (PPO) as a first scintillator, 1,4-Bis(5-phenyl-2-oxazolyl)benzene (POPOP) as a second scintillator, both dissolved in the mixture of toluene and Triton X-100) and the radioactivity was measured on scintillation counting instrument (Packard 1500 Tri-Carb Liquid Scintillation Analyzer).

Mass-spectroscopy on Glycated GPIIb/IIIa

Platelet membrane proteins were isolated as described in Assessment of glycation status of platelet membrane proteins from patients with the use of borohydride-[^{3}H].

Gel electrophoresis was applied before MS analysis to purify samples from proteases inhibitors that could prevent proper enzymatic digestion by trypsin. Selected protein bands excised from gel slices were crushed into small pieces and subjected to in-gel tryptic digestion. Briefly, the gel pieces were first de-stained in acetonitrile/50 mM NH_4HCO_3, pH 8.0, (1:1, *v*/*v*) and dehydrated in acetonitrile. The gel fragments were subjected to reduction with 10 mM dithiothreitol for 30 min at 56 °C and alkylation with 50 mM iodoacetamide for 45 min at 25 °C in the dark, followed by two alternating washing steps each with 25 mM NH_4HCO_3 and acetonitrile. Gel pieces were then dehydrated with acetonitrile, dried and subsequently rehydrated with a minimum volume of 25 mM NH_4HCO_3, pH 8.0, containing enough trypsin (sequencing grade, Promega) to provide a 1:10 trypsin-to-protein ratio. Incubation was performed overnight at 37 °C. After proteolysis, the supernatant was collected in PCR-tubes while gel pieces were subjected to two further extraction steps with 70 mL of 0.1% trifluoroacetic acid (TFA) in 2% acetonitrile. After centrifugation the supernatant was collected and applied for ESI-MS/MS analysis. NanoLC-ESI-MS/MS was performed on a Q-ToF mass spectrometer (Waters) coupled on-line to a nanoAcquity Ultra Performance LC (nanoAcquity UPLC) (Waters). For each measurement 3 mL of the digested sample was injected. Peptides were trapped on a trapping column C18, 180 mm × 20 mm, particle size 5 mm (Waters). The liquid chromatography separation was performed on a nanoACQUITY C18 column, 250 mm × 75 mm, particle size 1.7 mm (Waters) at a flow rate of 300 nl/min. The column temperature was set at 40 °C. Spectra were collected in the positive ion mode. The nanoflow ESI source conditions were set as follows: capillary voltage was set to 1850 V, sample cone voltage 40 V, extraction cone voltage 5 V, source temperature 80 °C. Data acquisition was controlled by MassLynx™ 4.1 software (Waters). The obtained MS/MS spectra were searched against SwissProt database with Protein Lynx Global Server Software (PLGS version 2.5.3 Waters Corporation, Milford, MA). Each sample of isolated membrane protein was run three times on LC-MS/MS. Early products of glycation (fructoselysine) were searched among all detected

peptides. Glycation which occurred at lysine which was first or last amino acid of the sequenced peptide were excluded from the studies. Only glycation of certain lysines, which were detected in three runs of the same samples, were taken into account. Coverage for integrin beta 3 was above 86% and for integrin alpha IIb above 54%. For each peptide at least 3 fragment ions were detected.

Statistical Analysis

We analysed the possible outliers with the use of either Grubb's or Tukey's tests. The missing data (if less often than 3% per a variable) were imputed using the *k* nearest neighbour analysis. The Student's t test for independent samples or Mann-Whitney's U test were used to compare the groups, depending on whether the data met the assumptions of data normality and homoscedasticity. The comparisons with the adjustments for dummy or confounding variables were performed using an analysis of covariance (ANCOVA) on raw or Box-Cox-transformed data. Simple correlations were estimated with the bootstrap-boosted rank Spearman correlation test, while the regression models were validated using the bootstrap-boosted procedures (1000–10,000 iterations). Simple correlations were estimated with rank Spearman correlation test (analyses for both T2DM and control groups), and the partial Spearman's rank test adjusted for group (analyses for all participating groups and separately for T2DM patients). Multiple group comparisons were performed with various models of the analysis of variance, followed by multiple comparisons post-hoc tests (Tukey's honest significant difference test). Canonical analysis was employed to determine which of the studied variables contributed to the highest extent to the significant association between selected sets of variables describing blood platelet activation and reactivity, blood platelet morphology and variables describing glycaemic control/protein nonenzymatic modifications. The aim was to build up a canonical (common) variable for each examined set of variables and to estimate the associations between various canonical variables (describing various sets of parameters); this would allow the examined variables contributing to the formation of the strongest associations between blood platelet functioning and metabolic control to be identified. As far as the statistical power of estimated differences/associations was often borderline, we used the resampling bootstrap technique (1000–10,000 iterations) to determine the likelihood of obtaining the revealed differences due to a pure chance (the bootstrap-boosted test statistics). To rank the predictors of the best discrimination between the groups ascribed as to either non-diabetic or diabetic patients, we employed a panel of classical statistical methods and data mining classifiers, including multiple logistic regression, linear discriminant analysis, canonical analysis, MAR Splines regression, support vector machine method, naïve Bayes classifier and *k* nearest neighbours (*kNN*) method. The outcomes of these analyses were further used to select the best predictors. The goodness of fit for the logistic regression models was evaluated using the Hosmer-Lemeshow test (Hosmer DW, Lemeshow S, Sturdivant RX. Applied Logistic Regression Third Edition Preface. Wiley Ser Probab St. 2013:Xiii-+).The heterogeneity of distributions of the flow cytometry variables referring to blood platelet size and the density of platelet surface membrane receptors was characterized with the use of STATISTICA Data Miner tool (the built-in algorithms of generalized expectation-maximization (EM) and k-means cluster analysis). This approach enabled us to decompose the overall distributions of platelet GPIIIa abundance or platelet size into two exponentially modified Gaussian (EMG) distributions of the componential platelet subpopulations. The resultant componential distributions were characterised by individual measures of central tendency and areas below the curves circumscribing given platelet subpopulations. For the purpose of this study, we decomposed overall distributions of platelet surface GPIIIa abundance (relevant to the anti-GPIIIa fluorescences) into two so-called partial distributions, which, from now on, we will refer to as ''subpopulation 1'' (with lower number of GPIIIa copies or smaller size) and ''subpopulation 2'' (with higher number of GPIIIa copies or bigger size).

Results

Glycation of Platelet Membrane Proteins

Platelets incubated in hyperglycemic conditions were glycated to higher extent (Me; IQR: 135.4; 123.7–171.7 vs. 302.9; 277.9–325.6 μM DMF for platelets incubated with Tyrode's vs. platelets incubated with glucose, respectively; $P < 0.0001$, $n = 15$).

Assessment of Blood Platelet Status As an effect of 4-day Incubation with Glucose or Mannitol

The fraction of fibrinogen-positive platelets (platelets bound exogenous fibrinogen) in control after 4-day incubation in PRP reached 47.5 ± 8.1% and significantly increased after TRAP stimulation (5 μM, 5 min) to 57.3 ± 8.9% ($P < 0.001$, $n = 16$). The similar tendency was observed for the samples incubated with glucose or mannitol, i.e., TRAP significantly elevated the fraction of fibrinogen-positive platelets. Neither glucose nor mannitol affected fibrinogen binding to platelets compared to control. Also, the fraction of CD62P-positive platelets did not differ between control and mannitol/glucose probes. Concentration of soluble P-selectin in incubates was significantly higher in mannitol and in glucose samples: 5.00 ± 3.65 ng/mL in control, 8.02 ± 3.74 ng/mL in glucose and 6.74 ± 3.58 ng/mL in mannitol ($P < 0.05$, $n = 16$). The mean volume of platelets (MPV) incubated with mannitol (8.0 ± 0.6 fl) or glucose (8.1 ± 0.8 fl) lowered compared to control (8.7 ± 0.7 fl) but this effect was not statistically significant.

Predictors of Platelet Functioning in Non-diabetic and Type 2 Diabetic Patients: Multivariate Analyses

A logistic regression analysis was performed to determine how selected analysed (confounding/co-explanatory) variables contribute to better discrimination between type 2 diabetic and non-diabetic individuals. The dependent variable was the dichotomised variable referred to as 'group' (type 2 diabetic, non-diabetic). The explanatory variables were those describing platelet function and those describing platelet glycation. For the purpose of this study the cross-validated model (cv) of logistic regression was employed. In the whole group, adjusted for sex and age, the most significant predictors of 'having diabetes' were the variables describing protein glycation: (OR_{GHb}^{cv} = 1.062 (95%CI: 0.810–1.408, $P < 0.04$), $OR_{fructosamine}^{cv}$ = 860 (95%CI: 24–30,520, $P < 0.0001$), $OR_{glycation}^{cv}$ = 2.610 (95%CI: 1.481–4.599, $P < 0.0010$, OR_{Glu}^{cv} = 11.66 (95%CI: 2.69–50.47, $P < 0.001$), $OR_{fructosamine}^{cv}$ = 860 (95%CI: 24–30,520, $P< 0.0001$)). Much lower 'discriminative power' was ascribed to the variables describing platelet function: $OR_{Fg\text{-}binding\ 2.5}^{cv}$ = 0.559 (95%CI: 0.395–0.792, $P < 0.001$), $OR_{plasma\ soluble\ GPIIIa}^{cv}$ = 1.001 (95%CI: 1.000–1.002, $P < 0.01$), $OR_{plasma\ soluble\ P\text{-}selectin}^{cv}$ = 0.536 (95%CI: 0.333–0.861, $P < 0.01$) and $OR_{GPIIIa\text{-}abundance}^{cv}$ = 0.99989 (95%CI: 0.99980–0.99999, $P < 0.03$). When constructing a multivariate model, the backward stepwise approach appeared the most convincing, as it resulted in the highest fraction of correct classifications (84.6%). Using this approach we revealed that upon the overall multiple *post hoc* standardization for sex and age, five variables: the activated GPIIb/IIIa expression in platelet agonized with 1 μM ADP, GPIIIa abundance in platelet surface membrane, soluble plasma GPIIIa, fibrinogen binding to platelets and small platelet microparticles appeared the significant predictors ($OR_{activated\ GPIIb/IIIa\ complex\text{-}ADP1,\ multivar}^{cv}$ = 1.106 (95%CI: 1.034–1.183, $P < 0.003$)), $OR_{GPIIIa\text{-}abundance,\ multivar}^{cv}$ = 0.99989 (95%CI: 0.99980–0.99999, $P < 0.01$), $OR_{plasma\ soluble\ GPIIIa,\ multivar}^{cv}$ = 1.002 (95%CI: 1.000–1.004, $P < 0.02$), $OR_{Fg\text{-}binding\ 2.5,\ multivar}$ = 0.598 (95%CI: 0.364–0.983, $P < 0.05$), $OR_{small\ microparticles,\ multivar}^{cv}$ = 0.172 (95%CI: 0.030–0.978, $P< 0.05$) ($P_{Hosmer\text{-}Lemeshow}$= 0.613).

To further ease the diagnosis of which variables are most useful in discriminating of the outcomes between non-diabetic and type 2 diabetic, we employed ROC analysis. Based in the calculated positive/negative true and positive/negative false scores, the ROC curve and the cut-off point were generated for each variable (with AUC values demonstrating the optimal sensitivity and specificity) and accuracy values determined ((true positive + true negative)/(true positive + true negative + false positive + false negative)). The highest AUC that reached statistical significance were revealed for GPIIIa abundance in platelet membranes (AUC = 0.641, $P < 0.05$), platelet binding to Fg (2.5 min) (AUC = 0.758, $P < 0.0001$), plasma soluble P-selectin (AUC = 0.687, $P < 0.006$) and plasma

soluble GPIIIa (AUC = 0.706, $P < 0.0002$). Likewise, the accuracy was the highest for $Fg_{binding\ 2.5}$ (0.867), soluble GPIIIa (0.684) and soluble P-selectin (0.661), GPIIIa abundance (0,651), activated GPIIb/IIIa expression in resting (0.633) and 1 μM ADP-activated platelets (0.617), platelet adhesion to Fg (0.615) and platelet microparticles (0.615).

Next, a linear discriminant analysis (LDA) was performed to determine, which of the analysed confounding/co-explanatory variables contribute the most to the discrimination between non-diabetic and type 2 diabetic individuals. The stepwise forward approach allowed to select these variables in the most reliable way. In general, based on the values of partial Wilk's lambda estimated upon adjustment for sex and age for all patients together, the most discriminative variables for non-diabetic and type 2 diabetic patients in the set of variables describing platelet functions appeared to be: GPIIIa abundance in platelet surface membrane ($\lambda_{partial\ Wilks} = 0.895$, $P < 0.015$), plasma soluble GPIIIa ($\lambda_{partial\ Wilks} = 0.903$, $P < 0.02$), the expression of the activated GPIIb/IIIa complex in 1 μM ADP-activated cells ($\lambda_{partial\ Wilks} = 0.915$, $P < 0.03$), Fg binding to platelets ($\lambda_{partial\ Wilks} = 0.925$, $P < 0.04$) and small platelet microparticles ($\lambda_{partial\ Wilks} = 0.918$, $P<0.035$), while glycated haemoglobin ($\lambda_{partial\ Wilks} = 0.658$, $P < 0.0001$), small microparticles ($\lambda_{partial\ Wilks} = 0.859$, $P < 0.004$), platelet protein glycation ($\lambda_{partial\ Wilks} = 0.882$, $P < 0.01$), GPIIIa abundance in platelet surface membrane ($\lambda_{partial\ Wilks} = 0.894$, $P < 0.015$) and the expression of the activated GPIIb/IIIa complex in 1 μM ADP-activated cells ($\lambda_{partial\ Wilks} = 0.944$, $P = 0.07$) remained most significant ones when pooling up variables of the set 1 and the set 2.

Canonical analysis was another approach undertaken to answer the question which of the studied variables contributed to the highest extent to the significant association between blood platelet function and the set of metabolic parameters linked to impaired carbohydrate metabolism. The idea was to build up a canonical (common) variable for each of the sets of variables and to estimate the associations between various canonical variables (describing various sets of variables) in order to figure out which of the examined variables contribute to the formation of the strongest associations. The set of platelet function variables consisted of the expression values of P-selectin and the activated GPIIb/IIIa complex recorded in resting platelets and platelets agonized with ADP or TRAP, GPIIIa abundance in platelet surface membranes, plasma soluble P-selectin and GPIIIa, Fg binding to platelets, platelet adhesion and platelet microparticles. The set of variables describing impaired glucose metabolism and glucose-mediated modifications of proteins included glycated haemoglobin, plasma fructosamine, platelet protein glycation and fasting glycaemia. We were interested to figure out which of the possible sets of independent (explanatory) variables (glycation markers) explains the maximal variability in the set of dependent variables (platelet function). Table 4 presents the canonical correlation coefficients between the canonical variable describing platelet function and the canonical variable describing platelet glycation. Obviously, the set 2, including the variables describing the effects of impaired glucose metabolism, was characterized by maximal values of extracted variance and elevated extents of redundancy (considerable co-linearity and low tolerance). Canonical correlation coefficients were high and very significant both for separate groups of type 2 diabetics and non-diabetics and for the pooled overall group of individuals studied. In the overall group the most significant contributors to the high canonical association between the sets were the plasma soluble forms of membrane glycoproteins, Fg binding to platelets, the expression of the activated GPIIb/IIIa complex in ADP-activated platelets, as well as glycated haemoglobin and hyperglycaemia. In turn, in a separate group of diabetic patients these contributors included Fg binding to platelets, the abundance of GPIIIa in surface membranes, glycation of platelet proteins and plasma fructosamine.

Finally, in order to determine which explanatory variables are the most significant predictors discriminating between subjects without and with type 2 diabetes mellitus we used a panel of multivariate data mining analysis (MAR Splines regression, support vector machine method, naïve Bayes classifier and *k* nearest neighbours (*kNN*)). The correctness of the outcomes of all the above-mentioned methods with a true (real) allocation to either group (non-diabetic or type 2 diabetic individuals) was evaluated with the use of the algorithm known as the voting of k judges. ROC curve analysis and calculated predictive values were used to assess which of the constructed models is most useful in differentiating between non-diabetic and type 2 diabetic patients and which combination of

parameters best discriminates between these two groups. Based on these calculations MARSpline (AUC = 1.000; correct classifications in 100% of non-diabetic and 100% type 2 diabetic individuals) and Bayes naïve estimator (AUC = 0.827; correct classifications in 70% of non-diabetic and 80% type 2 diabetic individuals) appeared the best techniques in discriminating non-diabetic and diabetic patients, while k-nearest neighbors (kNN) and support vectors methods revealed poorer correctness in the prediction (AUC < 0.760; correct classifications in 50-76% of non-diabetic and 62-85% type 2 diabetic individuals). The best predicting variables included Fg binding to platelets, the expressions of the activated GPIIb/IIIa complex and P-selectin in platelet surface membrane in response to low concentrations of ADP and TRAP, plasma soluble GPIIIa and P-selectin and platelet microparticles.

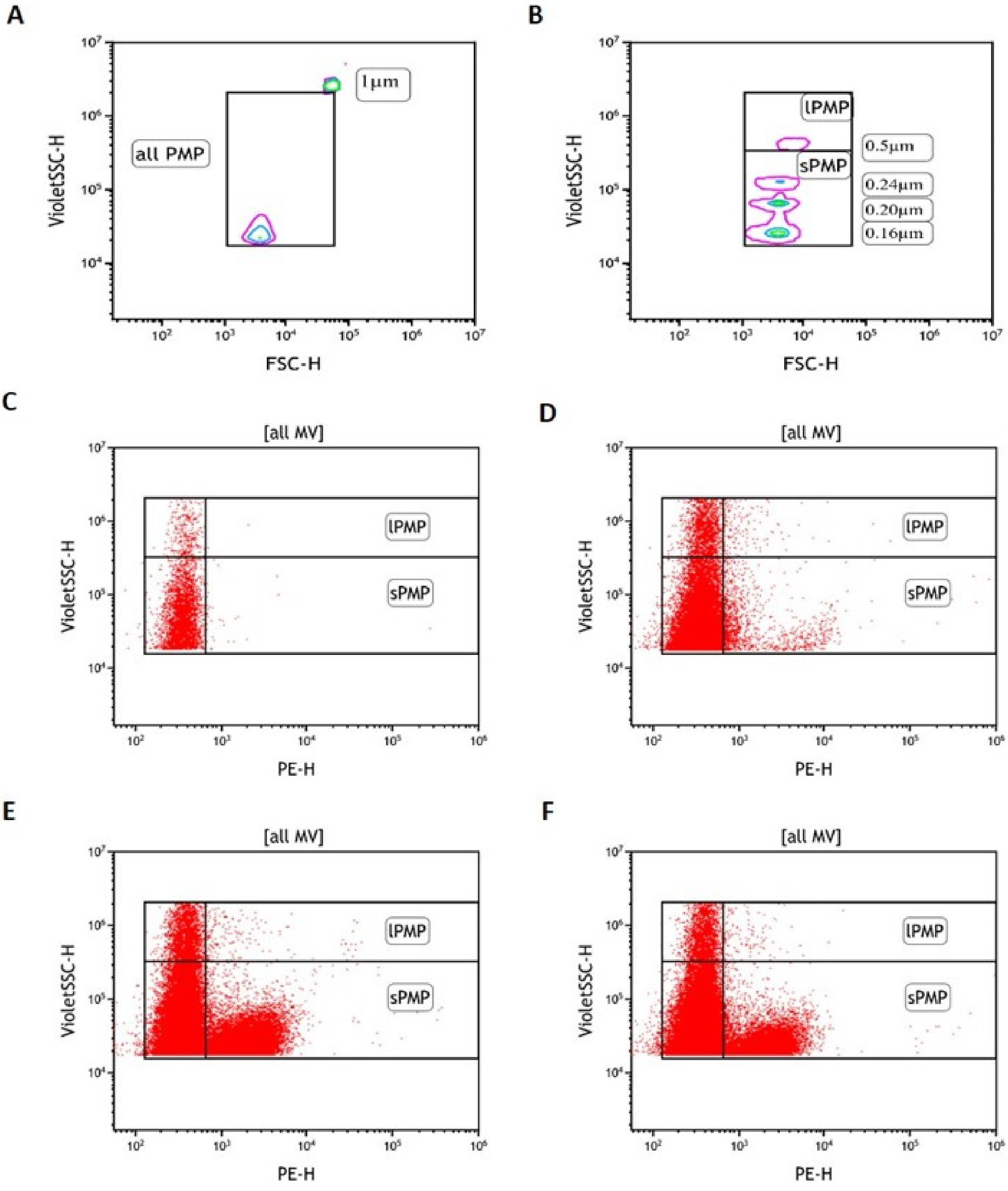

Figure S1. Exemplary dot plots of platelet derived microparticles (PMP) recorded using flow cytometry. Polystyrene beads of different sizes were used to determine the PMP gate (rectangular) between the 0.16 µm and 1 µm bead peaks (all PMP) (**A**). This gate was divided into the small PMP gate (sPMP) from 0.16 µm to 0.5 µm and the large PMP gate (lPMP) from 0.5 µm to 1 µm (**B**). The exemplary dot plots of microparticles were assayed in platelet-free-plasma samples obtained after

incubation of platelet-rich-plasma with Tyrode's buffer (**D**), 30 mM glucose (**E**) or 30 mM mannitol (**F**). Non-specific fluorescence was estimated according to the unstained sample (**C**).

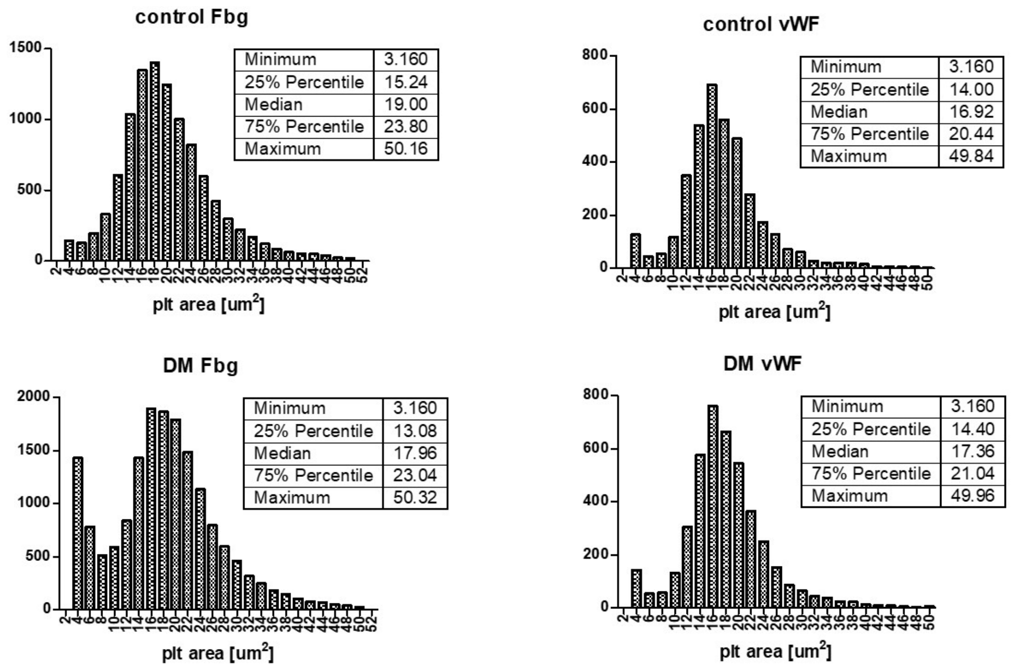

Figure S2. Platelets' area distribution. Histograms represent distributions of platelets' areas adhered according to the first protocol (whole blood diluted with autologous plasma). Data were accumulated from all samples in a given group (control or DM) run over an indicated protein.

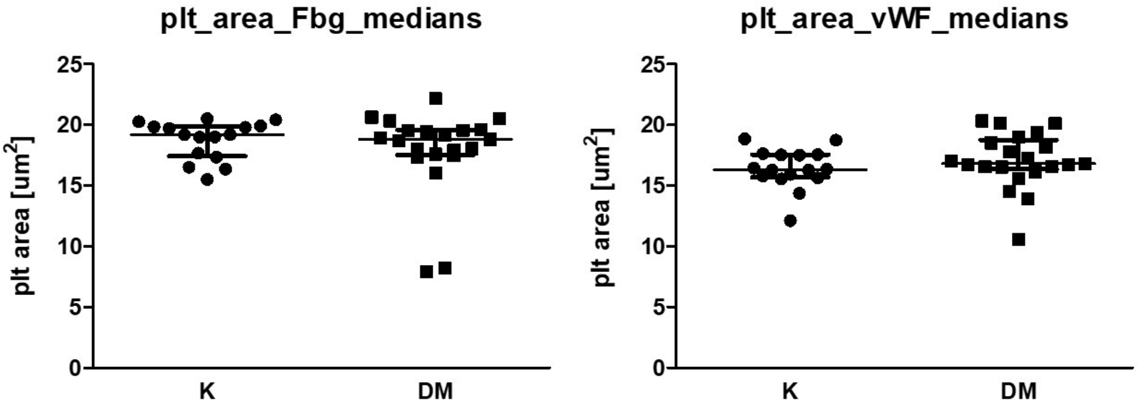

Figure S3. Areas of adhered platelets. Areas of individual blood platelets adhered according to the first protocol (whole blood diluted with autologous plasma). Each dot represents median area of platelet assessed in a given blood donor. Lines represent median with IQR.

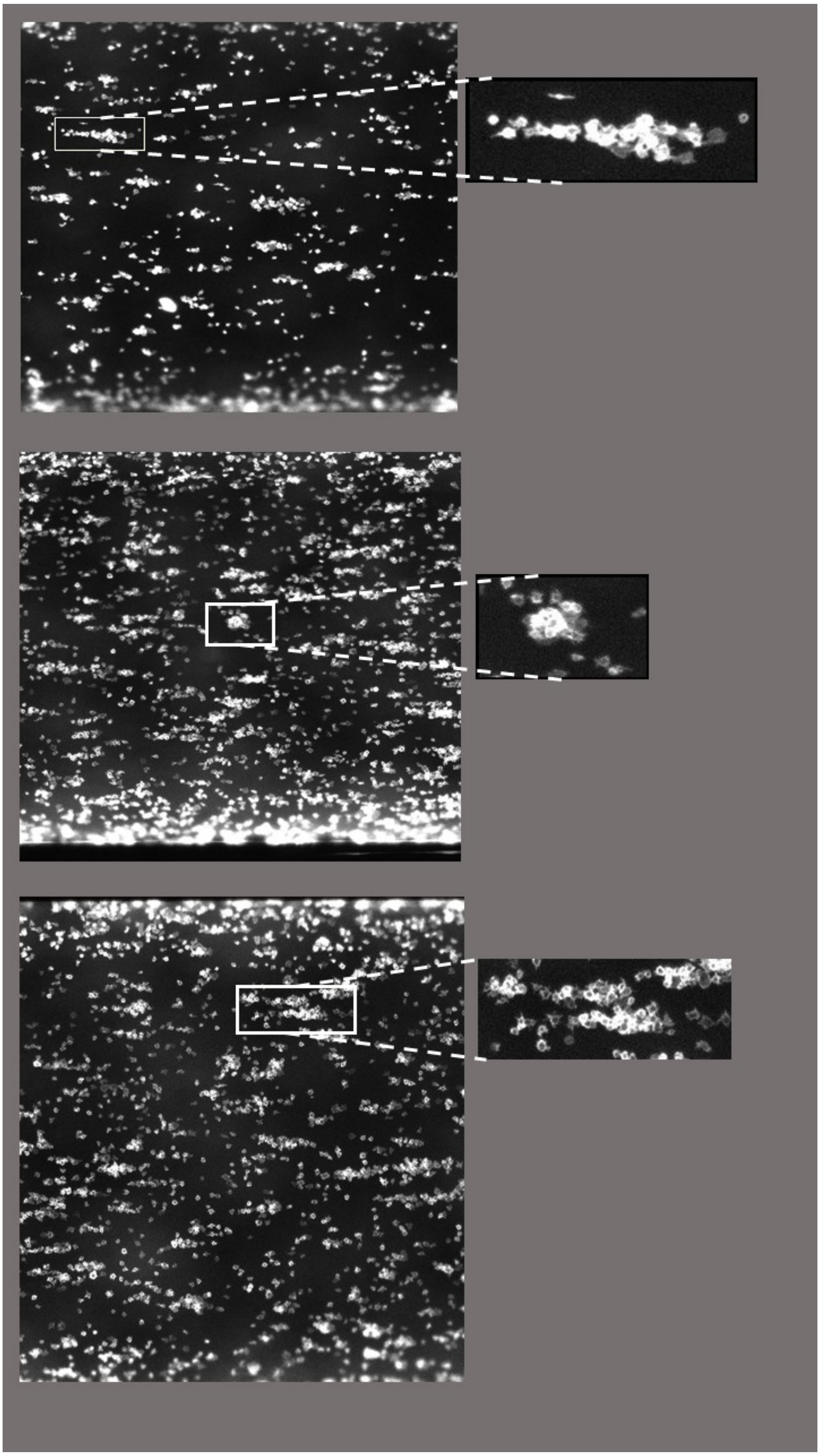

Figure S4. Exemplary images of the pattern of platelet adhesion in presence of 50% autologous RBC.

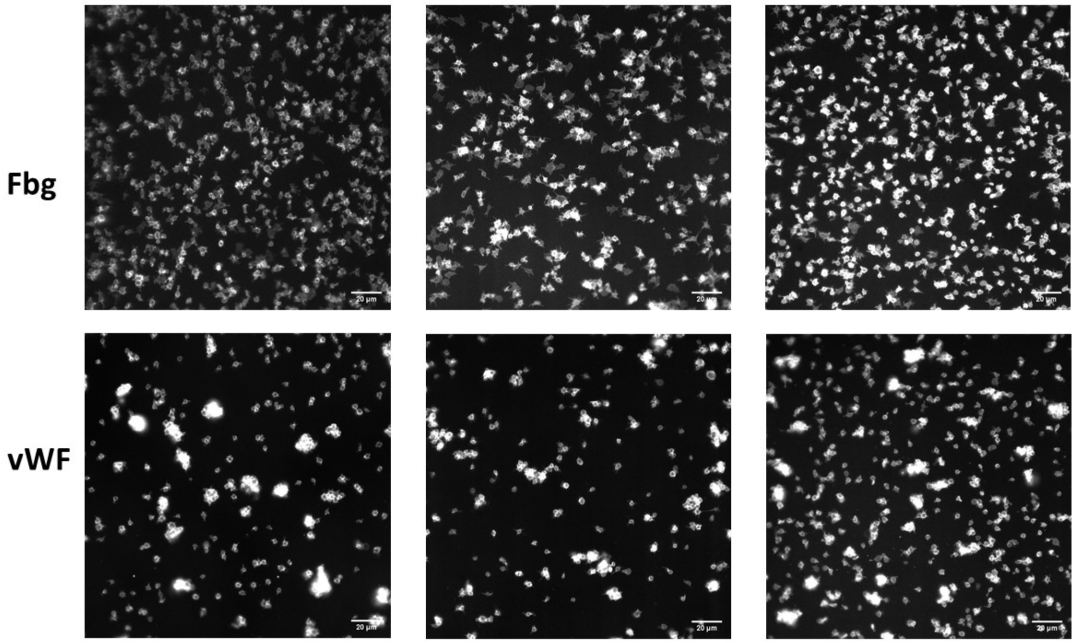

Figure S5. Exemplary images of the pattern of platelet adhesion in absence of PGE_1.

Table S1. Effects of in vitro incubation of platelet-reach plasma with glucose or mannitol on the fractions of GPIIIa-positive microparticles.

	Control (n = 16)	Glucose (n = 16)	Mannitol (n = 16)
small microparticles < 0.5 μm (μl^{-1})	27291 (10801; 89067)	49977 (23144; 124537)	46281 (35040; 83366)
large microparticles 0.5–1 μm (μl^{-1})	1654 (905; 2915)	1267 (740; 1580)*	1050 (565; 1698)#

Data presented as medians and interquartile ranges (Q1; Q3). Aliquots of platelet-rich plasma were incubated (4 days, 37 °C) with Tyrode's buffer solutions of 30 mM glucose or 30 mM mannitol with a continuous mixing on a wheel agitator. PRP samples treated only with Tyrode's buffer were used as the control. To determine the fractions of platelet-derived microparticles, PRP samples were centrifuged (12 min, 2000 × *g*), the resultant PPP samples were further centrifuged (5 min, 10 000 × *g*), stained with fluorolabelled MoAbs and further processed as described in details in the section *Materials and methods*. The significance of differences estimated with the bootstrap-boosted ANOVA on the Box-Cox-transformed data for the comparisons of control vs. glucose and control vs. mannitol: * $P < 0.05$; # $P < 0.05$.

Table S2. Canonical correlations between the set of blood platelet reactivity parameters and the sets of protein glycation parameters.

Platelet Reactivity (Set 1)	Extracted Variance (%)	Total Redundance (%)	Explanatory Variables (Set 2)	Extracted Variance (%)	Total Redundance (%)	Canonical Correlation	Canonical Determination (R^2)	*P*	Wilks' Lambda	Best Contributors
both groups	33.2	10.5	*protein glycation*	100.0	34.3	0.721	0.520	0.003	0.268	plasma soluble sGPIIIa, plasma soluble sP-selectin, $Fg_{binding\ 2.5}$, activated form of GPIIbIIIa $_{ADP1}$, GHb, Glu
type 2 diabetic pts	45.4	22.1	*protein glycation*	100.0	48.7	0.823	0.677	0.0001	0.101	$Fg_{binding\ 2.5}$, $GPIIIa_{ab}$, glycation, fructosamine
control subjects	34.4	16.15	*protein glycation*	100.0	42.1	0.818	0.669	0.0001	0.080	$adhesion_{Fg}$, activated GPIIbIIIa $_{ADP1}$, glycation, GHb

The set 1 of variables included: the abundance of GPIIIa in platelet surface membranes, the expression of the activated GPIIb/IIIa complex in resting platelets and upon stimulation with either 1 or 10 μM ADP, the expression of P-selectin in resting platelets and upon stimulation with either 1 or 10 μM ADP, the soluble form of GPIIIa in blood plasma, the soluble form of P-selectin in blood plasma, the bonding of fibrinogen to whole blood platelets for 2.5–5–7.5 min, small (<500 nm) and large (500–1000 nm) platelet microparticles, platelet adhesion to fibrinogen; the set 2 of variables included: glycaemia, glycated haemoglobin HbA_{1c}, plasma fructosamine, glycation of platelet (membrane) proteins. When analysing the associations in separate non-diabetic or type 2 diabetic groups, we used the resampling with replacement (bootstrap-boosted analysis) adjusted for the overall sample size (n = 65), Rho^2 is a squared canonical correlation coefficient (canonical determination), which is relevant to variance between canonical variables. Total redundancy is relevant to the variance between the canonical variable for set 1 and the variables of set 2; it shows the representation of a given canonical variable (set 1) by the explanatory variables of the set 2. Extracted variance is a variance between a given canonical variable and the variables that build it up; it reflects how well a given canonical variable represents a given set of variables (how redundant are the variables of the set 2 for the variables of the set 1). The Wilks' lambda is the parameter reflecting the contribution of a set 2 to the explaining of the variance of a set 1; the lower is the Wilks' lambda, the higher is the contribution. ***Abbreviations*:** activated GPIIbIIIa $_{ADP1}$, expression of the activated GPIIb/IIIa complex in platelet surface membranes in response to 1 μM ADP; $adhesion_{Fg}$, platelet adhesion to fibrinogen; $Fg_{binding\ 2.5}$, binding of fibrinogen to whole blood platelets for 2.5 min; fructosamine, plasma fructosamine; GHb, glycated haemoglobin, Glu, overnight fasting glycaemia; glycation, non-enzymatic glycosylation of platelet proteins; GPIIIa $_{ab}$, abundance of GPIIIa in platelet surface membrane; HbA_{1c}; sGPIIIa, plasma soluble GPIIIa; sP-selectin, plasma soluble P-selectin.
